# Development and characterisation of improved unifocal primary mouse lung cancer models with metastatic potential

**DOI:** 10.1002/path.6435

**Published:** 2025-06-18

**Authors:** Ana‐Rita Pedrosa, Alejandro Castillo‐Kauil, Yuliia Kravchuk, Louise Reynolds, Bruce Williams, David Moore, Cameron Lang, Srinivas Allanki, Eleni Maniati, Alexandros Hardas, Jozafina Haj, Rebecca Drake, Julie Cleaver, Julie Foster, Jana Kim, Ester Stern, Jane Sosabowski, Gilbert O. Fruhwirth, Erik Sahai, Ori Wald, Kairbaan Hodivala‐Dilke

**Affiliations:** ^1^ Adhesion and Angiogenesis Lab, Centre for Tumour Microenvironment, John Vane Science Centre, Barts Cancer Institute Queen Mary University London UK; ^2^ Department of Pathology University of Cambridge Cambridge UK; ^3^ Department of Pathology CRUK Lung Cancer Centre of Excellence, UCL Cancer Institute London UK; ^4^ Imaging Therapies and Cancer, Comprehensive Cancer Centre, School of Cancer and Pharmaceutical Sciences, Faculty of Life Sciences and Medicine King's College London London UK; ^5^ Tumour Cell Biology Lab The Francis Crick Institute London UK; ^6^ Centre for Cancer Evolution, John Vane Science Centre, Barts Cancer Institute Queen Mary University London UK; ^7^ Royal Veterinary College Hertfordshire UK; ^8^ Francis Crick Institute London UK; ^9^ Cancer Imaging Lab, Centre for Cancer Biomarkers and Biotherapeutics, John Vane Science Centre, Barts Cancer Institute Queen Mary University London UK; ^10^ Department of Cardiothoracic Surgery, Hadassah Medical Center and Faculty of Medicine Hebrew University Jerusalem Israel

**Keywords:** Unifocal, non‐small cell lung cancer (NSCLC) preclinical mouse models, metastasis, lung cancer, histopathological features, SPECT/CT, sodium iodide symporter, matrix remodelling, immune infiltration

## Abstract

Lung cancer is the leading cause of cancer‐related death globally. To better understand the biology of lung cancer, mouse models have been developed using either tail vein‐injected tumour cell lines or genetically modified mice. The current gold‐standard models typically present with multiple lung foci. However, although these models are widely used, their correlation with human disease are limited, as early‐stage human lung cancer usually presents as a single lesion rather than multiple foci. Additionally, a major challenge of using multifocal lung tumour models is the difficulty in distinguishing primary lung tumours from intrathoracic metastasis and lethal levels of lung congestion before distant metastases develop. Here, we present a refined and detailed surgical method in which murine tumour cells [Lewis lung carcinoma (LLC), alveogenic lung carcinoma (CMT), or *Kras/Trp53‐*KP mutant cells] were injected directly into the left lung lobe of C57BL/6 mice, or, alternatively, adenoviral‐Cre or adenoviral‐FlpO was administered directly into the left lung lobe of *Kras* ^*LSL‐G12D*^;*Trp53* ^*fl/fl*^ or *Kras* ^*FSF‐G12D*^;*Trp53* ^*frt/frt*^ (KP) mice, respectively. This method generated unifocal primary left lung lobe tumours with traceable spread to local and distant sites. A cross‐comparison of the unifocal models described commonalties and differences between LLC, CMT, KP cells, and adenoviral‐Cre or ‐FlpO methods in terms of timings for primary lung tumour growth and traceable spread to local and distant sites, histological analysis of CD3 and CD11b immune cell infiltration, and Picrosirius Red analysis of extracellular matrix complexity. Lastly, the frequency of clinical histopathological features typical of human lung cancer were assessed across the unifocal mouse models to provide a direct comparison with human lung cancer. Overall, this study details a refined and reproducible protocol for intralobular lung injection to generate unifocal lung cancer models that resemble key features of human lung cancer. This approach can be applied to other lung cancer initiation strategies. The cross‐comparative histological analysis across the models tested here offers a valuable resource to aid researchers in selecting the most appropriate next‐generation unifocal lung cancer models for their specific research needs. © 2025 The Author(s). *The Journal of Pathology* published by John Wiley & Sons Ltd on behalf of The Pathological Society of Great Britain and Ireland.

## Introduction

Lung cancer is the leading cause of cancer deaths worldwide. In 2020, estimates suggested that more than 1.7 million people died of lung cancer worldwide. Early‐stage lung cancer presents most commonly as a single primary tumour, and more advanced disease involves metastasis to contralateral lung lobes, lymph nodes, liver, adrenal glands, and brain, among other sites [1]. Current strategies to treat lung cancer include a combination of surgical resection and, where appropriate, chemotherapy, radiotherapy, targeted therapy, and immunotherapy [2, 3]. To accelerate the development of treatment strategies, improved models of lung cancer that faithfully display the combination of genetic and pathological features of the human disease *in situ* would be advantageous.

Current experimental models for studying the causal effects of lung cancer progression and resistance to therapy range from two‐dimensional (2D) and three‐dimensional (3D) cell‐based systems to *in vivo* growth of lung tumour cells and genetically modified mouse models (GEM) of lung cancer [4, 5, 6, 7]. Inducible models, generated by intratracheal administration of a virus‐based Cre‐ or FlpO‐recombinase in GEM, such as KP or KPL (*Kras* ^*LSL‐G12D*^;*Trp53* ^*fl/f*^; *Lkb1* ^*fl/fl*^), enable the generation of cancer specifically in lung tissue, and provide temporal control over genetic events within a specific lung cell population [8, 9, 10]. However, unlike human lung cancer, which usually presents with a single primary tumour, most *in vivo* models develop multiple lung tumour foci, which limits clinical relevance. The assessment of *bone fide* primary tumours versus intrathoracic metastases is also difficult to assess. Moreover, multifocal models develop a high tumour burden in the lungs, necessitating the culling of the mice and often present with pre‐invasive, multiple foci at an early stage, making it equally challenging to study invasive advanced disease with substantial stromal response. More recently, GEM models have been used with very low viral titres administered intratracheally to generate individual tumour nodules in the lungs [11]. However, this approach results in long latency periods, with no obvious ability to distinguish between the primary tumour and subsequent lesions.

Here we describe a detailed surgical method for the development of unifocal primary lung tumours either by injection of tumour cells into the left lung of mice or by injection of adenoviral‐Cre or adenoviral‐Flippase (FlpO) into the left lung of *Kras* ^*LSL‐G12D*^;*Trp53* ^*fl/fl*^ or *Kras* ^*FSF‐G12D*^;*Trp53* ^*frt/frt*^ (KP) mice respectively. We have expanded on previously published work [12, 13] to demonstrate that: (1) different mouse lung tumour cell lines can be used to replicate different lung cancer phenotypes that share similarities with those observed in human disease and (2) adenoviral delivery into KP GEM mouse models initiates lung tumour formation that closely reflects multiple features of human lung cancer. Furthermore, by analysing the different murine unifocal models for tumour progression, metastasis formation, immune cell infiltration, extracellular matrix (ECM) composition, and comparison with human pathological features, we have created a useful resource to help researchers select the most appropriate preclinical model for their specific research purposes.

## Materials and methods

### Ethical approval

All animal studies were approved by Queen Mary University London Animal Ethics Committee licensed under the UK Home Office regulations and the Guidance for the Operation of Animals (Scientific Procedures) Act 1986 (Home Office, London, UK), including Amendment Regulations 2012 and UK Coordinating Committee on Cancer Research Guidelines for the Welfare and Use of Animals in Cancer Research.

### Mice

*FSF‐Kras* ^*G12D*^;*Trp53* ^*frt/frt*^ and *LSL‐Kras* ^*G12D*^;*Trp53* ^*fl/fl*^ strains and the C57BL/6 mouse strain were used. All animals were aged between 8 and 12 weeks at the start of the experiments, and both male and female mice were used in all experimental cohorts. Further details on housing conditions are provided in Supplementary materials and methods.

### Virus preparation

Matrigel was thawed at 4 °C overnight and stock virus vials were thawed on ice on the day of the experiment. Each mouse received 2.5 × 10^7^ PFU of adenovirus‐FlpO (Ad5CMVFlpO, VVC‐U of Iowa 530, University of Iowa, Iowa, USA) or 1.25 × 10^7^ PFU of either luciferase‐tagged adenovirus‐Cre (Ad5CMVCre‐P2A‐FFLuciferase, VVC‐U of Iowa‐8,194‐1,000) or adenovirus‐Cre (Ad5CMVCre, VVC‐U of Iowa‐5) in a final volume of 30 μl (standard concentration Matrigel, Catalogue No.: 356234, Corning, Ewloe, Flintshire, UK). Alternatively, each mouse received in a final volume of 10 μl (high concentration Matrigel, Catalogue No.: 354263) Minimum essential medium (MEM, Catalogue No.: 21090–022, Gibco, Thermo Fisher Scientific, Altrincham, Cheshire, UK)/Matrigel containing either 1.25 × 10^7^ PFU of adenovirus‐Cre (Ad5CMVCre, VVC‐U of Iowa‐5) or 0.8 × 10^7^ PFU of luciferase‐tagged adenovirus‐Cre (Ad5CMVCre‐P2A‐FFLuciferase, VVC‐U of Iowa‐8,194‐1,000). MEM was prepared by adding 2 mm L‐glutamine and 12 mm CaCl_2_. Virus was added to 15 or 5 μl modified MEM and mixed by inverting the tube, for 30 or 10 μl injections respectively. Once prepared, virus mixture was stored at room temperature before intralobular injection for a maximum of 2 h. An equal volume of Matrigel was added to the virus mixture and mixed carefully to avoid bubbles.

For intratracheal administration each mouse receives 2.5 × 10^7^ PFU of luciferase tagged adenoviral‐Cre in a final volume of 60 μl in MEM solution.

### Tumour cell preparation

The following non‐small cell lung cancer (NSCLC) cell lines were used: KP cells (KPB6 RRID:CVCL_C0RJ), a kind gift from Julian Downward's lab, derived from *LSL‐Kras* ^*G12D*^;*Trp53* ^*fl/fl*^ strain; Lewis lung carcinoma (LLC) cells (3LL RRID:CVCL_5653), Catalogue No.: CRL‐1642 (ATCC, Glasgow, Scotland, UK); CMT167 cell line (CMT167 RRID:CVCL_2405), Catalogue No.: 151448 (Ximbio, Stratford, London, England, UK). Cell lines were mycoplasma tested (in‐house). For culture conditions, see Supplementary materials and methods.

After culturing and collection, cells were resuspended in an appropriate amount of PBS as per calculations (for LLC, each mouse receives 1 × 10^4^ cells in 30‐μl injections or 3,300 cells in 10 μl; for CMT and KP, each mouse receives 1 × 10^4^ cells in 30‐μl injections). An equal volume of Matrigel was added to the cell suspension and mixed carefully by inverting the tube to reduce the formation of bubbles. Cells were kept on ice prior to injections.

### Whole genome sequencing and murine sodium iodide symporter (mNIS) [14, 15, 16, 17] of cell lines

Further details on the generation of *in vivo* traceable mouse lung cancer cell lines are provided in supplementary material, Figure S1. Data are also available at: https://www.ncbi.nlm.nih.gov/bioproject/PRJNA1220686.

### Thoracotomy procedure

In brief, mice were anaesthetised and laid on their right side, and the preshaved left thoracic area was sterilised. The ribcage was identified and a vertical incision made on the mid‐point between the ribcage and the shoulder. Subcutaneous tissue was removed to expose the pleura with the ribcage and left lung lobe underneath. A preloaded syringe was then immediately inserted at a depth of 5 mm and the mixture dispensed. A cotton bud was applied to the place of injection before slowly removing the syringe. The two skin flaps were then brought together and two wound clips applied. The mouse was then laid on its left side to recover from anaesthesia. Further details are provided in Supplementary materials and methods (see the step‐by‐step protocol) and Figure S2 and Table S1.

### Intratracheal administration of adenoviral‐Cre in KP GEM mice

Mice were anaesthetised i.p. with an injectable anaesthetic solution (xylazine with ketamine) and MEM viral mixture administered directly into the trachea, as previously described [18]. Further details are provided in Supplementary materials and methods.

### SPECT/CT imaging and respiratory gated lung CT

Mice with mNIS (mouse sodium iodide symporter) cell‐induced left lung tumours were injected via tail vein injection with approximately 30 MBq Tc99m‐pertechnetate (Technetium 99, Barts Health Radiopharmacy, Barts Health NHS Trust, London, UK) in 200 μl PBS. Following a 50‐min uptake period, the mice were anaesthetised using isoflurane maintained at 300 ml/min and 2% oxygen and placed in a heated imaging bed. Respiration and anaesthesia were monitored and adjusted if necessary to maintain the breathing rate between 40 and 60 breaths per min. A 45‐min SPECT scan was acquired at 1 h after injection followed by a 5‐min whole‐body CT scan using a VECTor6‐CTxuhr SPECT/CT scanner (MILabs Utrecht, Houten, the Netherlands). For respiratory gated lung CT, an ultra‐focus respiratory gated thorax CT was acquired using the same CT instrument. Further details on the SPECT and CT imaging parameters and equipment setup are available in Supplementary materials and methods.

### IVIS imaging

Mice were injected i.p. with luciferin at a dose of 150 mg/kg from a 30‐mg/ml luciferin stock solution (VivoGlo Luciferin‐ Firefly Luciferase substrate Promega, Catalogue No.: P1043, Madison, WI, USA) 10 min before culling. Once culled, the mouse lungs were inflated with 800 μl of luciferin *ex vivo*, removed, and transferred to a Petri dish. Both dorsal and ventral images of the lungs were acquired with an IVIS machine (IVIS Lumina Series 3, PerkinElmer, Waltham, MA, USA) with an exposure time of 5 min (300 s). Details on tissue collection and histological analysis are provided in Supplementary materials and methods.

### Statistical analyses

Prism software version 10 (Irvine, CA, USA) was used for all statistical analyses. Specific details on each statistical analysis are provided in the corresponding figure legends.

## Results

### Unifocal left lung lobe tumours induced by adenoviral‐FlpO in KP GEM mice and using KP, CMT, and LLC cells in C57BL/6 mice and their metastasis patterns

**Figure 1:**
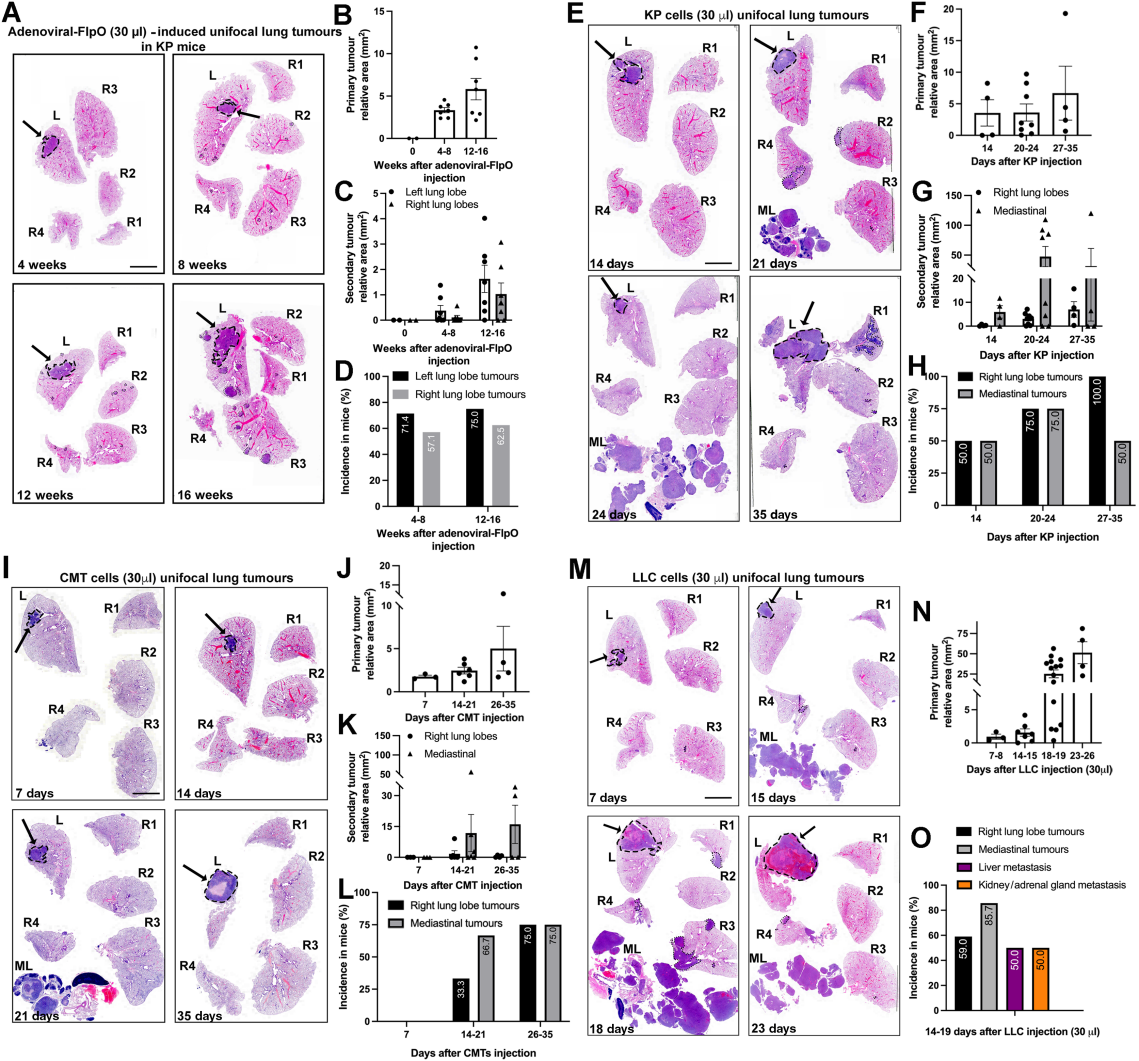
Unifocal lung tumours induced by adenoviral‐FlpO in KP GEM mice or cells injected into C57Bl6 mice. (A) Representative whole‐lung H&E staining at different time points after adenoviral‐FlpO injection in KP GEM mice. *Dashed black lines* and *arrows* identify primary tumour foci in left lung lobes. *Dotted black lines* denote right lung lobe tumours. (B and C) Bar graphs denote primary tumour relative area and secondary tumours relative area (from H&E image, mm^2^) in time course after adenovirus injection, respectively. Each dot represents a single mouse. (D) Bar graph denoting incidence in mice of right lung lobe tumours (expressed as a percentage of total mice at each time point after injection). *n* = 2 mice at 0 weeks; *n* = 7 mice at 4–8 weeks; *n* = 7 mice at 12–16 weeks. (E, I, and M) Representative H&E‐stained sections of lungs at time points after KP, CMT and LLC cells 30 μl injection respectively. *Dashed black lines* and arrows highlight primary tumour foci in left lung lobes. *Dotted black lines* denote right lung lobe tumours. *ML*, mediastinal lymph nodes with presence of tumour. (F, J, and N) Bar graphs denoting primary tumour relative area in time courses after KP, CMT, LLC 30‐μl cell injection respectively. (G and K) Bar graphs denoting secondary right lung lobe and mediastinal tumours relative area (from H&E‐stained sections, mm^2^) in time courses after KP and CMT 30‐μl cell injection. Each dot represents a single mouse. (H and L) Bar graphs denoting incidence in mice of right lung lobe tumours (black bars) and mediastinal tumours (grey bars) (expressed as a percentage of total mice at each time point after injection) in KP and CMT injected models. (O) Bar graphs denoting incidence in mice of right lung lobe tumours (black bars), mediastinal tumours (grey bars), liver metastasis (purple bars), and kidney/adrenal gland metastases (orange bars), expressed as a percentage of total mice after injection. For KP injected mice, *n* = 4 mice at 14 days; *n* = 8 mice at 20–24 days; *n* = 4 mice at 27–35 days. For CMT injected mice, *n* = 3 mice at 7 days; *n* = 6 mice at 14–21 days; *n* = 4 mice at 26–35 days; For LLC injected mice (30 μl), *n* = 3 mice at 7–8 days; *n* = 7 mice at 14–15 days; *n* = 15 mice at 18–19 days; *n* = 4 mice at 23–26 days. Scale bar, 4 mm.

Analysis of lungs at 4, 8, 12, and 16 weeks following injection of adenoviral‐FlpO into the left lung lobe of KP GEM mice revealed progressive growth of primary tumours in the left lung lobe (supplementary material, Figure S2). A high percentage of mice (>50% of mice 8 weeks after injection onwards) also presented with both left lung lobe secondary tumours and contralateral right lung lobe tumours. However, no mediastinal lymph node tumours were detected in this mouse model (Figure 1A–D and supplementary material, Figure S3A).

As expected, injection of KP, CMT, and LLC (10,000 cells in 30 μl) NSCLC cell lines, with relevant common mutations found in human disease (supplementary material, Figure S1A,B, supplementary material, Table S3), into the left lung lobe of C57BL/6 mice developed clearly defined primary tumours at a faster rate than in KP GEM mice (Figure 1E–O, and supplementary material, Figure S3B–D).

KP and CMT tumour growth rates were similar, with both developing well‐defined primary tumours in the left lung lobe from 7 to 14 days after inoculation (Figure 1E–L and supplementary material, Figure S3B,C). Both models developed contralateral right lung and mediastinal tumours (Figure 1E,G–I,K,L, and supplementary material, Figure S3B,C). Injection of LLC cells also generated well‐defined primary tumours in the left lung lobe from 7 days after inoculation (Figure 1M,N, and supplementary material, Figure S3D). By approximately 20 days after LLC inoculation, mice had to be culled due to high tumour burden in the thoracic cavity, and most presented with secondary tumours in right lung lobes and mediastinal lymph nodes. Notably, half the mice also exhibited liver and kidney and/or adrenal gland metastases (Figure 1O).

### Longitudinal *in vivo* SPECT/CT imaging can be used to track the progression of left lung lobe primary tumour growth in intralobular, unifocal lung cancer models

**Figure 2:**
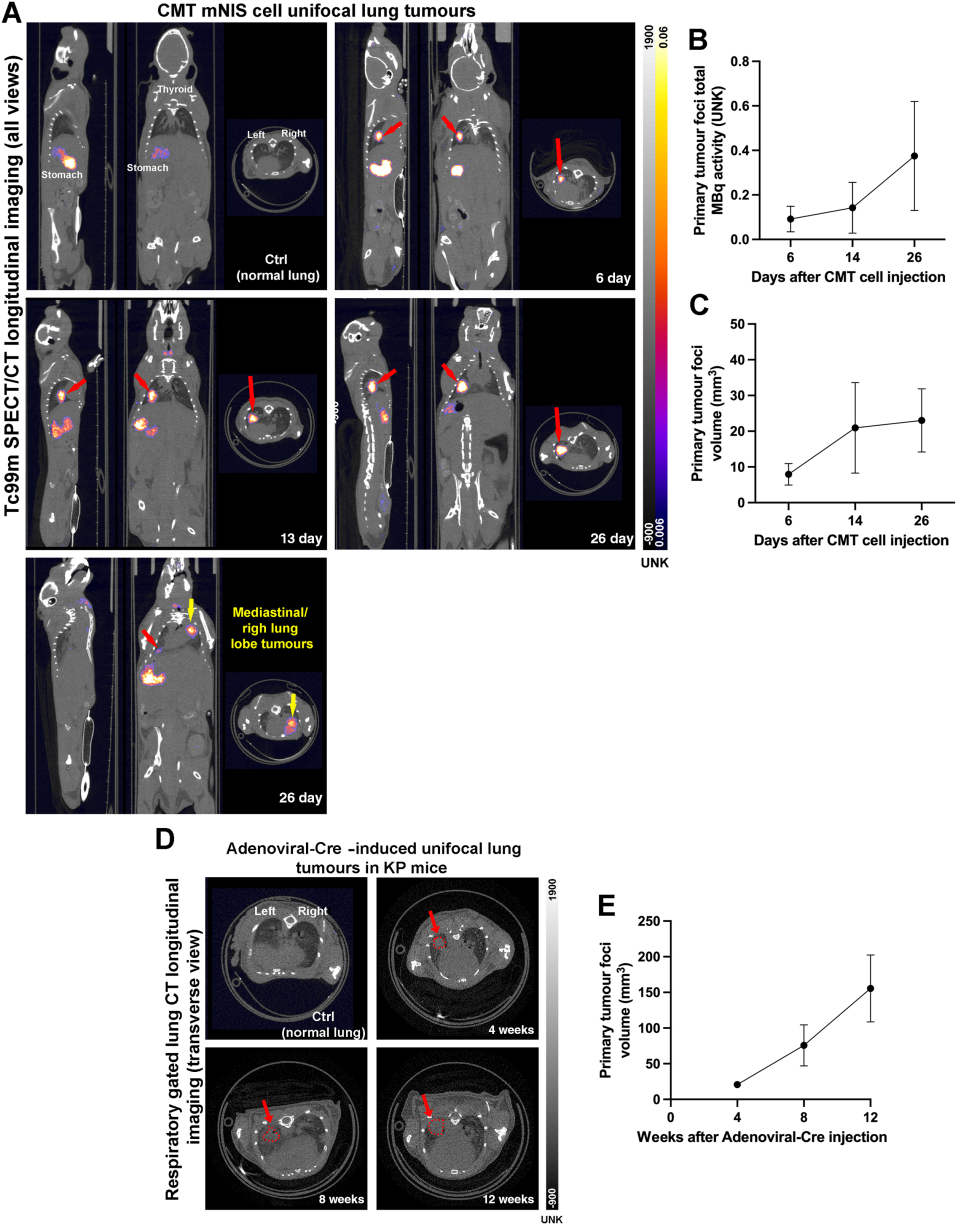
*In vivo* SPECT/CT longitudinal imaging to track growth of left lung lobe primary tumour and metastasis development in unifocal lung intralobular models. (A) Representative SPECT/CT overlaid images (all views) of progressive time points after CMT murine sodium iodide symporter (mNIS) cell injection into left lung lobe. Top left quadrant: control mouse (not injected with cells; Ctrl) with no Tc99m uptake in lungs (signal from stomach, thyroid, and bladder constitutes normal physiological uptake of radionuclide in these organs). *Red arrows* highlight primary tumour injection site in the left lung lobe with high Tc99m uptake signal that increases over time. Bottom left quadrant: mouse with right lung and/or mediastinal tumours highlighted by yellow arrow. Please note that the image is taken in plane to visualise the mediastinal tumour that is not in the same plane as the primary left lung lobe tumour. *Scale bars* for both CT and non‐calibrated SPECT signals are shown on the right of the panel. (B) Primary tumour total MBq activity line graph represents total amount of Tc99m activity (UNK) in total volume of primary tumour, over time. (C) Primary tumour volume line graph represents total volume (mm^3^) increase over time. *n* = 4 mice/time point (6, 14, and 26 days). (D) Representative respiratory gated ultra‐focus thorax CT images (transversal view) of progressive time points after adenoviral‐Cre left lung lobe injection into KP GEM mice. Top left quadrant: control mouse (not injected with virus; Ctrl) denoting normal structures of lungs. *Red arrows* and dashed lines highlight primary tumour foci in left lung lobe that increases over time. (E) Primary tumour volume line graph represents total volume (mm^3^) increase over time. *n* = 5 mice/time point (4, 8, and 12 weeks).

To illustrate the application of intralobular lung cancer models for longitudinal imaging, mouse NSCLC cell lines (CMT and KP cells) expressing mNIS [19] (supplementary material, Figure S1C–E) were used for radiolabelled *in vivo* tracking of left lung lobe primary tumours by single‐photon emission computed tomography (SPECT) (Figure 2 and supplementary material, Figure S4).

Increased radioactivity (MBq) in the left lung lobe was detected from 6 to 26 days after tumour cell injection, as unifocal primary tumours grew (Figure 2A–C and supplementary material, Figure S4). Additionally, right lung lobe or mediastinal tumours were also detected by the presence of radioactivity outside the primary injected site at 26 days after injection (lower SPECT panel in Figure 2A). Of note, radioactivity signal levels detected in the thyroid, stomach, and bladder were physiologically normal, as epithelial cells from these tissues naturally uptake Tc99m‐pertechnetate due to endogenous expression of mNIS [20].

Ultra‐focus respiratory gated thorax CT also allowed *in vivo* tracking of adenoviral‐Cre‐induced left lung tumours in KP GEM. Left lung lobe tumours could be detected as early as 4 weeks after adenoviral injection, and progressive growth was measurable in terms of tumour volume (Figure 2D,E).

Therefore, the models presented here allow high‐quality analysis and tracking of tumour development *in vivo*, which also allows better accuracy in planning treatment interventions in preclinical studies.

### Refined injection conditions to reduce possible pleural fluid leakage from injected primary site

Luciferase‐tagged adenoviral‐Cre was injected intralobularly into the left lung of KP GEM mice using a 30‐μl volume in standard concentration Matrigel (1.25 × 10^7^ PFU as described above). *Ex vivo* luminescence analysis showed that 82% of mice had signal only in the left lung lobe (supplementary material, Figure S5A,B). Notably, left lung lobe tumour luminescence increased progressively up to 2 weeks after injection. No luminescence was detected at 4 weeks after injection due to expected luciferase dilution as the tumours grew (supplementary material, Figure S5A,C,D).

**Figure 3:**
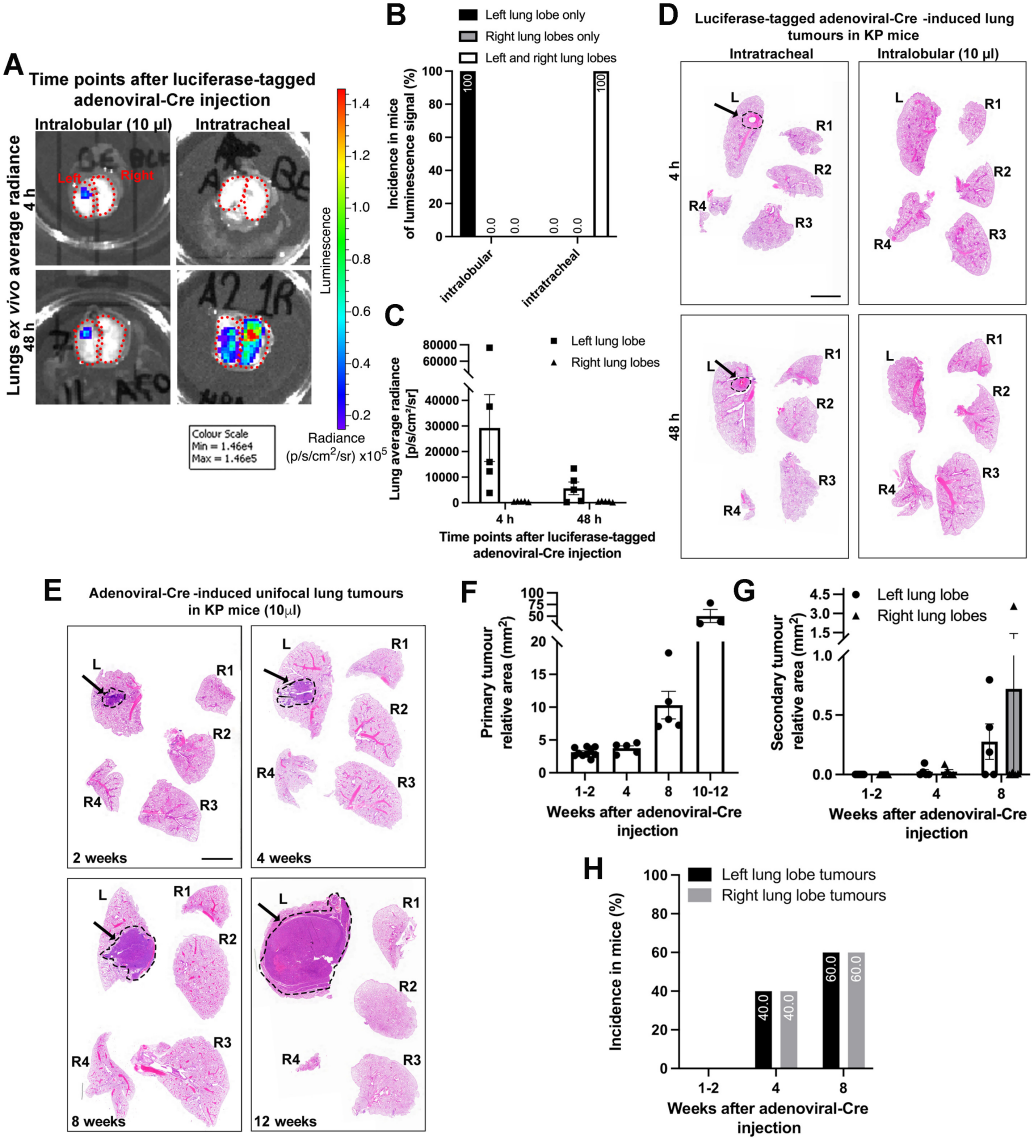
Refined unifocal lung tumours induced by adenoviral‐Cre in KP GEM mice (using 10 μl volumes with high‐concentration Matrigel). (A) Luciferase‐tagged adenoviral‐Cre was either injected intralobularly into the left lung lobe of KP GEM mice to generate a single primary tumour focus (0.8 × 10^7^ PFU with high concentration Matrigel in 10‐μl injection volume) or administered intratracheally (1.25 × 10^7^ PFU with MEM in 60‐μl injection volume). Mice were culled and lungs imaged *ex vivo* using IVIS at 2–4 and 48 h after injection to assess leakage from primary tumour injection site. Representative images of lung dorsal view radiance at different time points after injection. *Dotted red lines* represent left and right lung lobes and intensity of radiance is seen in lungs according to scale on right. (B) Bar graph denoting incidence of luminescence signal detected in left lung lobe alone, right lung lobe alone, or both (considering both dorsal and ventral lung views), expressed as a percentage of total mice, combining the acute time points, 4 and 48 h (and only including mice that had signal present). *n =* 3 mice, intratracheal and *n* = 8 mice, intralobular. (C) Bar graph denotes left lung lobe average radiance (p/s/cm^2^/sr) calculated by summing the dorsal view radiance and ventral view radiance in left lung lobe for intralobular model. Each dot represents a mouse. *n =* 5 mice for 4 h; *n* = 5 mice for 48 h. (D and E) Representative H&E‐stained sections of lungs at (D) 4 and 48 h time points after adenoviral‐Cre intralobular injection or intratracheal administration, and (E) 2, 4, 8, and 12 weeks, after intralobular injection. *Dashed black lines* and *black arrows* highlight injected site in left lung lobes (D). (F and G) Bar graphs denote primary tumour relative area and secondary tumours relative area (from H&E image, mm^2^) in time course after adenovirus injection. Each dot represents a single mouse. Scale bar, 4 mm. (H) Bar graph denoting incidence of mice that presented right lung lobe tumours (expressed as a percentage of total mice at each time point after injection, excluding the 10‐ to 12‐week time points, as mice had primary tumours that encompassed the whole left lung lobe). *n* = 10 mice at 1–2 weeks; *n* = 5 mice at 4 weeks; *n* = 5 mice at 8 weeks; *n* = 3 mice at 10–12 weeks.

To refine this model further, luciferase‐tagged adenoviral‐Cre was injected intralobularly into the left lung of KP GEM mice using a 10‐μl injection volume in high‐concentration Matrigel (0.8 × 10^7^ PFU) (Figure 3). Notably, *ex vivo* lung luminescence revealed that 100% of mice had signal present only in the left lung lobe at acute time points (4 and 48 h) after injection. As expected, this was in contrast with intratracheally administered luciferase‐tagged adenoviral‐Cre (2.5 × 10^7^ PFU in 60 μl volumes in MEM), which resulted in signal in both left and right lung lobes of 100% of the mice (Figure 3A–D). Compared with the previous injection conditions (Figure 1A–D), this refined approach showed the development of slower growing primary tumours and a slight reduction in the incidence of left and right lung secondary tumours that were much smaller in size (Figure 3E–H).

**Figure 4:**
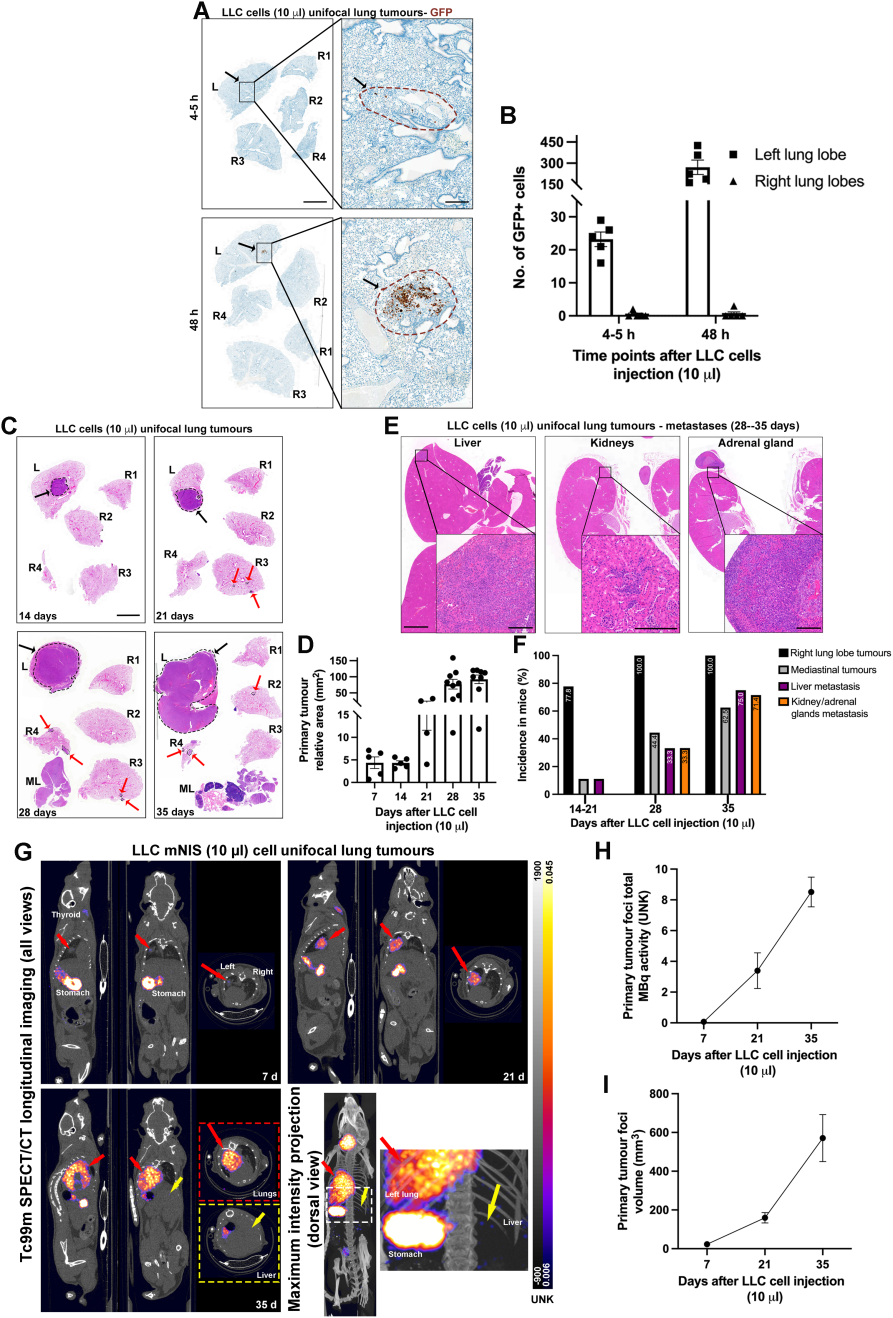
Refined unifocal lung tumours induced by LLC cells injected into C57Bl6 mice using 10‐μl volumes with high‐concentration Matrigel. (A) Representative immunohistochemistry of GFP (DAB with haematoxylin blue counterstain), in lungs at acute time points (4 and 48 h) after LLC murine sodium iodide symporter (mNIS) GFP injection (3,300 cells in 10‐μl volumes with high‐concentration Matrigel). *Black arrows*, *black boxes*, and *dashed brown lines* denote injection site, which is shown in higher magnification on the right. *Scale bar*, 4 mm at low magnification and 0.4 mm at high magnification. (B) Bar graph showing number of GFP‐positive cells present in lungs. Each dot represents values for a single mouse. Data given as mean ± SEM. (C and E) Representative H&E‐stained sections of lungs, and metastatic organs, at different time points after 10‐μl injection of LLC cells. *Dashed black lines* and *black arrows* highlight primary tumour foci in left lung lobes; *dotted black lines* and *red arrows* denote secondary right lung lobe tumours; *ML*, mediastinal lymph nodes with presence of tumour. *Scale bars*, 4 mm for lungs in panel (C), 2 mm for organs at low magnification, and 0.2 mm for organs at high magnification in panel (E). (D) Bar graphs denoting primary tumour relative area in time courses after LLC 10‐μl cell injection. Each dot represents values for a single mouse. Data given as mean ± SEM. (F) Bar graphs denoting incidence in mice of right lung lobe tumours (black bars), mediastinal tumours (grey bars), liver metastasis (purple bars), and kidney and adrenal gland metastasis (orange bars), expressed as a percentage of total mice after injection, in time course after 10‐μl LLC injection; *n* = 5 mice at 4–5 h, *n* = 5 mice at 48 h, *n* = 7 mice at 7 days, *n* = 5 mice at 14 days, *n* = 4 mice at 21 days, *n* = 9 mice at 28 days and *n* = 8 mice at 35 days. (G) Representative SPECT/CT overlaid images (all views) of progressive time points after LLC mNIS cell injection (10 μl) into left lung lobe (signal from stomach, thyroid, and bladder constitutes normal physiological uptake of radionuclide in these organs). *Red arrows* and *red dashed square* highlight primary tumour foci in left lung lobe with high Tc99m uptake signal that increases over time. *Yellow arrows* and *yellow dashed square* highlight extra‐thoracic metastasis with transverse view at level of liver. Bottom right quadrant shows a maximum‐intensity projection with dorsal view and amplified region denoting by a *yellow arrow* several small hot spots at level of liver. Scale bars for both CT and non‐calibrated SPECT signals are shown on right of panel. (H) Primary tumour total MBq activity line graph represents total amount of Tc99m activity (UNK) in total volume of primary tumour, over time. (I) Primary tumour volume line graph represents total volume (mm^3^) increase over time. *n* = 8 mice at 7 days and 21 days, *n* = 3 mice at 35 days.

The same refined approach was tested using the highly metastatic and fast developing LLC cell line by injecting 3,300 LLC cells in 10‐μl volume high concentration Matrigel into C57BL/6 mice. At 5–6 and 48 h after injection, immunohistochemistry revealed the presence of GFP+ LLC cells only in the left lung lobe, at the injection site (Figure 4A,B). Up to 35 days after injection, primary tumours continued to develop in the left lung lobe and showed a slower increased incidence of mediastinal tumours (Figure 4C,D,F,G,I) compared with mice injected with LCC (in 30 μl) (Figure 1M). Importantly, mice still presented with *bona fide* extra‐thoracic metastasis to the liver, kidney, and/or adrenal glands, as seen by both H&E stained sections and SPECT/CT analysis (Figure 4E–G and supplementary material, Movies [Link], [Link]). We also observed lymphoid hyperplasia due to extramedullary haematopoiesis in the spleen and liver (data not shown). Notably, we did not observe brain metastasis within this cohort. However, the same 10‐μl approach with standard‐concentration Matrigel led to a small percentage of mice (two out of six mice) also presenting brain metastasis in the subarachnoid space (supplementary material, Figure S6A–C).

These observations suggest that using refined conditions (reduced injection volumes with high‐concentration Matrigel) correlates with a reduction of secondary intrathoracic dissemination and mediastinal tumours. However, this refined method allows for primary tumours to grow for longer and increased incidence of extra‐thoracic metastasis to the liver, kidneys, and adrenal glands.

### Comparison of lymphocytic and myeloid immune cell infiltration across unifocal NSCLC models

**Figure 5:**
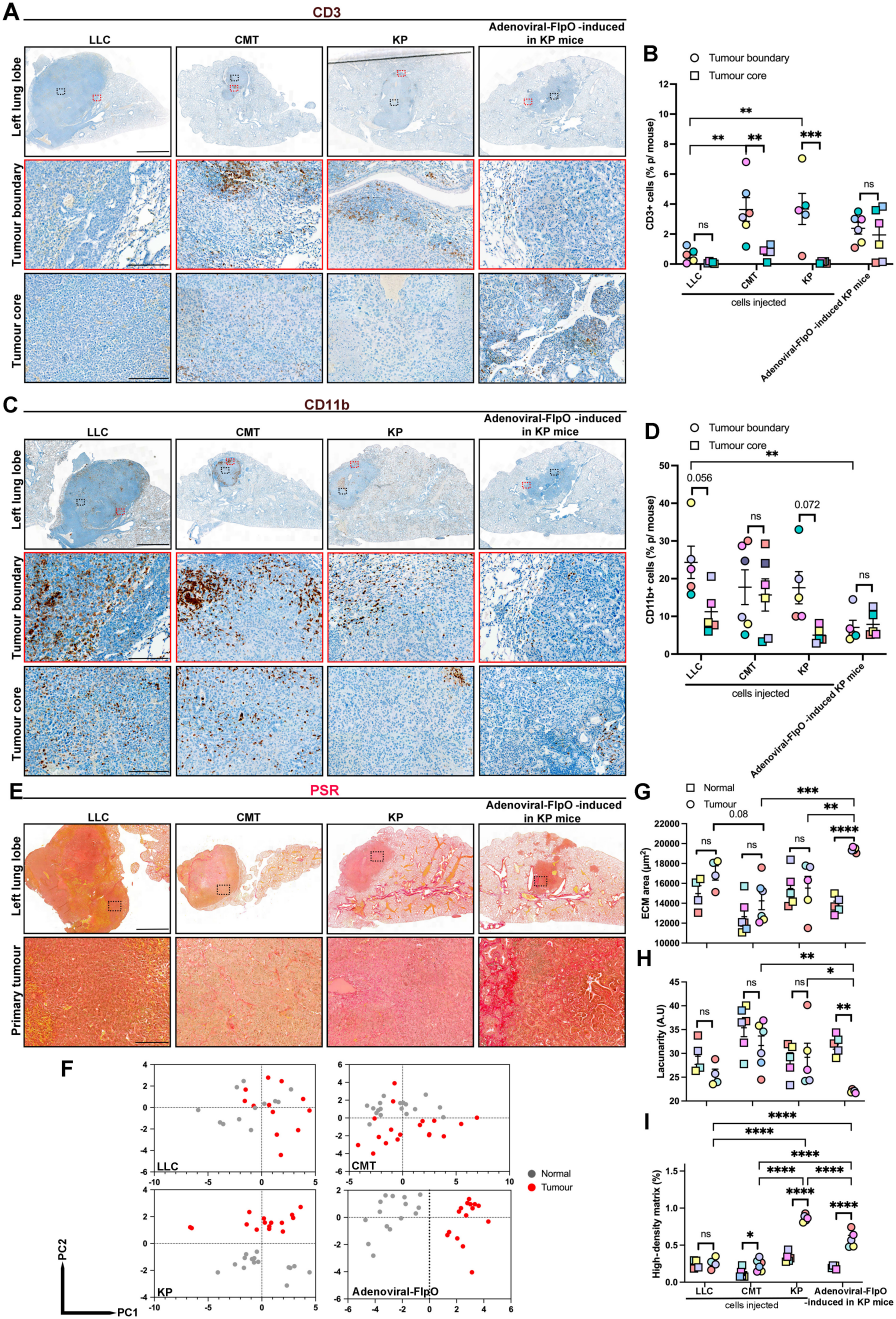
Characterisation of lymphocytic and myeloid immune cell infiltration and ECM pattern of normal and tumour tissue in unifocal lung intralobular models. (A and C) Representative immunohistochemistry images of CD3 and CD11b (DAB with haematoxylin blue counterstain) respectively of primary left lung unifocal tumours (in unifocal LLC, CMT, and KP cells and adenoviral‐FlpO‐induced tumours in KP GEM mice). Upper row: low‐magnification images. *Black dashed boxes*, tumour core; *red dashed boxes*, tumour boundary and tumour core presented at higher magnification in lowers rows. (B and D) Scatter dot plots showing percentage of CD3‐ and CD11b‐positive cells, respectively, in each intralobular model. Each dot represents a mouse. Data given as mean ± SEM. LLC, *n* = 5 mice; CMT, *n* = 6 mice; KP, *n* = 5 mice; adenoviral‐FlpO in KP mice, *n* = 6 mice (only mice with established tumours were used). (E) Representative immunohistochemistry images of Picrosirius Red (PSR in red and Weigert's haematoxylin in brown/orange) of primary left lung unifocal tumours, denoting higher‐magnification images (*dashed rectangles* show location of higher magnification images in left lung lobe lower magnification images). (F) PCA plots show overall ECM architecture in lung tumours (red dots) and their corresponding normal regions (grey dots) in LLC, CMT, KP, and adenoviral‐FlpO in KP mice models. (G–I). Dot plots showing measurements: (G) ECM area (in μm^2^), (H) Lacunarity (measure of how ECM fills space in arbitrary units), and (I) high‐density matrix (as percentage) in tumour compared with normal tissue. Each dot represents one mouse. LLC, *n* = 4 mice; CMT, *n* = 6 mice; KP, *n* = 5 mice; adenoviral‐FlpO in KP mice, *n* = 5 mice (only mice with established tumours were used). Two‐way ANOVA with Tukey's multiple comparisons test was used. *ns*, not significant; *p ≤ 0.05; **p ≤ 0.01; ***p ≤ 0.001; ****p ≤ 0.001). *P* values are also given as numerical values for some comparison. Scale bars, 2 mm at lower magnification (upper panels), 0.2 mm at higher magnification (middle and bottom panels).

With increasing interest in the mechanisms underpinning improved immunotherapy treatment of NSCLC [21, 22, 23], a baseline overview of immune cell infiltration (lymphocytic‐CD3 and myeloid‐CD11b markers) was performed to compare across the unifocal models tested (Figure 5A–D). These were also compared with commonly used subcutaneous tumours for cell lines and the multifocal intratracheally induced adenovirus‐based model (supplementary material, Figure S7A–D).

Overall, the mouse unifocal models described generate varied CD3 immune cell responses, ranging from almost complete absence of lymphoid cells in the LLC model to relatively high infiltration in the CMT and KP models (mainly retained at the tumour boundary) (Figure 5A,B). Notably, the unifocal adenovirus‐based model showed a higher percentage of infiltrating CD3^+^ cells in the tumour core compared with the conventional multifocal model (supplementary material, Figure S7A,B). These observations are also consistent with the genetic signatures identified for these cell lines (supplementary material, Figure S1A). *Stkb11* mutations (or *Lkb1*, observed only in LLC cells) are associated with poor T‐cell infiltration, whereas *Trp53* mutations (loss‐of‐function only in KP cells) are associated with higher T‐cell infiltration in NSCLC [24].

Unifocal cell line‐derived models generated a considerable myeloid reaction, whereas adenovirus‐generated tumours in KP mice did not (Figure 5C,D). A similar profile was observed in matched subcutaneous and multifocal models. However, in general, for the cell lines (except for LLC), these models presented higher infiltration of myeloid cells retained at the boundary and lower infiltration in the tumour cores (supplementary material, Figure S7C,D).

Overall, these data highlight the inability of multifocal models to generate adequate adaptive and innate immune responses.

### Unifocal lung intralobular models show diverse remodelling of extracellular matrix

Lung sections were stained with Picrosirius Red to examine ECM organisation in both primary unifocal tumours and surrounding normal tissue (Figure 5E–I) and in matched subcutaneous and multifocal models (supplementary material, Figure S7E–I). TWOMBLI (The Workflow Of Matrix Biology Informatics) image analysis was performed (Supplementary material, Table S2) [25].

Principal component analysis (PCA) on all output parameters from TWOMBLI showed that the overall ECM architecture in LLC and CMT unifocal tumours was similar in tumour and adjacent normal tissue. In contrast, ECM architecture parameters clustered separately from their normal adjacent tissue in KP cell‐injected lung tumours and adenoviral‐FlpO‐induced lung tumours in KP mice (Figure 5F). In subcutaneous models, PCA showed greater similarity between CMT and KP tumours than with the LLC model. Interestingly, in multifocal adenovirus‐generated tumours, adjacent normal tissue and tumour also did not differ in overall ECM architecture (supplementary material, Figure S7F).

For adenoviral‐FlpO unifocal induced tumours, ECM area was significantly higher and lacunarity inversely lower in the tumour compared with normal tissue (Figure 5G,H). Significant differences in high‐density matrix between normal and tumour were higher in both KP cell and adenovirus‐induced tumours compared to other models (Figure 5I). In matched subcutaneous and multifocal models (supplementary material, Figure S7G–I), CMT and KP cell tumours showed the highest levels of ECM area and high‐density matrix and, conversely, the lowest levels of lacunarity. Of note, and unlike the unifocal model, the adenoviral‐based multifocal model showed no difference or even lower ECM area and high‐density matrix, indicating a complete lack of ECM remodelling.

In brief, cell‐based subcutaneous models show more ECM compared with unifocal lung tumours. However, adenoviral induced unifocal tumours in KP mice presented a distinct tumour desmoplastic reaction reminiscent of human NSCLC disease, which was not observed in multifocal matched models.

### Preclinical mouse unifocal lung intralobular models present histopathological features resembling human NSCLC disease

**Figure 6:**
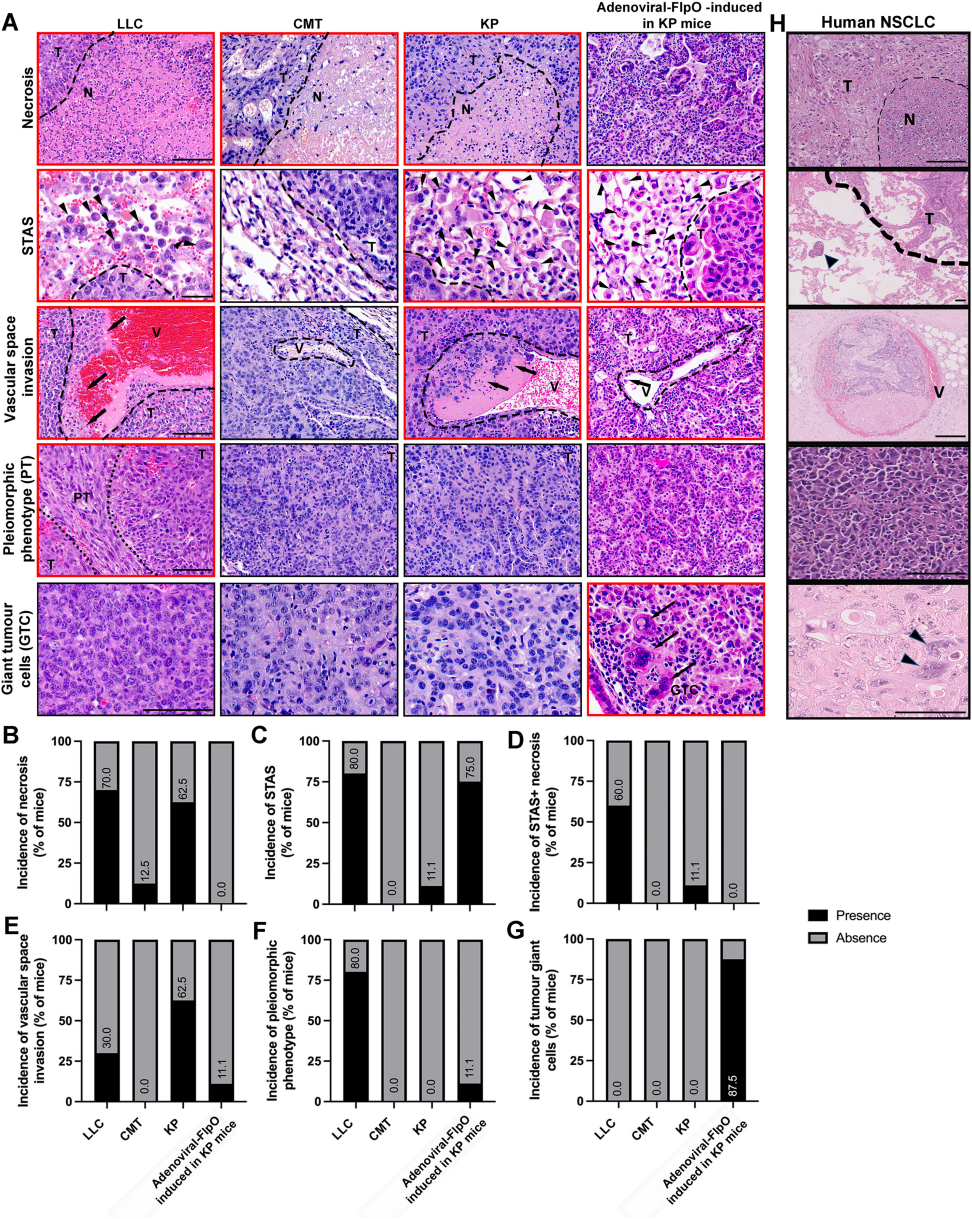
Comparison of clinical histopathological features of human NSCLC with mouse unifocal lung intralobular models. (A) Representative H&E images of necrosis, STAS, vascular space invasion, pleiomorphic phenotype (PT), and GTC histopathological features in mouse unifocal left lung lobe intralobular models (LLC, CMT, KP cells, and adenoviral‐FlpO in KP mice). Images with red borders show respective features, whereas images with black boxes lack specific histopathological features. *Black dashed lines* denote separation of tumour areas [T; necrotic areas (N); boundary or limit of tumour (in STAS images); separation of tumour and vessels (V); and separation of PT from non‐pleiomorphic areas (T)]. *Black arrowheads* in STAS images represent presence of tumour cells in 1 or more airspaces beyond the boundary of the tumour; *long black arrows* represent tumour cell invasion into the vasculature. *Scale bars*, 0.1 mm in first, third and fourth row panels; 40 μm in second row panels. (B–G) Incidence of histopathological features expressed as a percentage of total mice, in established tumours (in LLC, CMT, KP cells, and adenoviral‐FlpO in KP mice). *n =* 10 mice for LLC; *n* = 8 for CMT; *n* = 8 for KP; *n* = 8 for adenoviral‐FlpO‐induced tumours in KP mice. (H) Representative H&E images of necrosis, STAS, vascular space invasion, PT, and GTC histopathological features in human primary NSCLC samples.

Human NSCLC displays common histopathological features which are used in the diagnosis of the disease. The mouse unifocal models described here were thus analysed for signs of such histopathological features [necrosis, spread through air spaces (STAS), vascular space invasion, pleiomorphic phenotype (PT), and presence of giant tumour cells (GTCs)] in comparison with human NSCLC (Figure 6A,H).

In early‐stage NSCLC, necrosis has been associated with poor prognosis, reduced overall survival, and disease‐free survival and commonly increases in patients after neoadjuvant therapy [26, 27]. While necrosis was present in a high percentage of mice in LLC and KP cell models, it was absent in adenoviral‐FlpO‐induced KP tumours (Figure 6A,B), suggesting slower disease progression.

STAS is defined as micropapillary clusters, solid nests, or solitary cells spreading through air spaces beyond the edge of the primary tumour [28, 29]. It is also a feature that is associated with poorer prognosis, as it relates to local recurrence and an invasion pattern typical of adenocarcinomas (ADCs) [30]. STAS was observed in the LLC and adenoviral‐FlpO‐induced models and at a low percentage of mice in the KP cell‐injected model (Figure 6A,C). STAS is also commonly associated with necrosis and lymphovascular invasion [30]. Of note, the presence of necrosis and STAS was mainly observed in the LLC model and in around 10% of mice in the KP cell‐injected model (Figure 6A,D).

Vascular space invasion, or intratumoral vascular invasion, is a hallmark of metastatic spread in all solid cancer types. Accordingly, in stage I NSCLC, it has been associated with increased risk of cancer‐specific mortality [31], associated with a worse overall survival in patients with resected NSCLC [32] and a significant risk factor for recurrence [33]. Vascular space invasion was observed in a high percentage of mice in the KP cell‐injected model and in a lower percentage in LLC and adenoviral‐FlpO‐induced KP mice (Figure 6A,E).

Pleiomorphic carcinoma is defined as a poorly differentiated rare type of NSCLC, which includes epithelial components comprising squamous cell carcinoma (SqCC), ADC, or undifferentiated NSCLC, which displays at least 10% spindle and/or giant cells or a carcinoma consisting only of spindle and giant cells. Pleiomorphic carcinoma has a poor prognosis even in early‐stage NSCLC [34, 35]. Strikingly, a pleiomorphic phenotype was observed in 80% of LLC tumours (Figure 6A,F), where more than 10% of the primary tumours consisted of spindle‐shaped cells (as shown in the H&E as PT, in Figure 6A). Occasional giant tumour cells were observed in the adenoviral induced model (Figure 6A,G).

In summary, the unifocal LLC model presented features consistent with very aggressive tumours, such as high incidence of necrosis, STAS, vascular invasion, and pleiomorphic characteristics, which was in agreement with its description as a highly tumourigenic, metastatic and resistant model to several anticancer treatments [36]. This was consistent with the co‐occurring mutations of *Kras* and *Stk11* (or *Lkb1*) observed in this cell line (supplementary material, Figure S1A, supplementary material, Table S3), as these have been associated with early metastatic dissemination and adenosquamous subtype in human NSCLC [24]. Furthermore, of the three cell lines tested, LLC is the one with more copy number alterations, highlighting its diverse mutational landscape and high genomic instability (supplementary material, Figure S1B). The KP cell model also presented aggressive features, such as necrosis and vascular invasion, which were reduced in CMT tumours. This also aligned with evidence suggesting that *PiK3ca* mutations, present in LLC and KP but not in CMT cells (supplementary material, Figure S1A), are encountered preferentially in advanced‐stage tumours [24]. Conversely, *Rb1* mutations (observed only in CMT cells) were associated with poorer outcomes in NSCLC (supplementary material, Table S3), possibly consistent with its metastatic origin. The adenoviral‐FlpO‐induced KP tumours presented a mixed phenotype, with no necrosis and low incidence of vascular invasion or pleiomorphic phenotype, but with STAS and presence of occasional GTC, highlighting a slower and progressive tumour development, characteristic of GEM models.

Overall, these data identify various histopathological similarities between preclinical unifocal lung models and human disease, highlighting their potential applications in preclinical lung cancer research.

## Discussion

The first description of a lung cancer unifocal protocol dates back to 1999, in which LLC cells implanted directly into the lung parenchyma of C57BL6 mice led to the development of mediastinal lymph node metastasis [12]. Subsequent studies examined the tumorigenic and lymph node metastatic potential of human NSCLC cell lines [13, 37, 38]. Furthermore, unifocal lung cancer in GEM models using adenoviral‐Cre was first described by Herter‐Sprie *et al* [39]. Despite these initial publications, the methods were not described in detail and were not standardised or optimised across various models, and their relevance to human lung cancer clinicopathological features were lacking.

Based on previous methodologies, we presented a refined study with a step‐by‐step description of the optimised surgical orthotopic left lung lobe implantation of mouse NSCLC cell lines in C57BL/6 mice or adenoviral‐Cre or adenoviral‐FlpO in KP GEM mice. The objective of our study was to provide a method that could be easily reproduced and streamlined across the lung cancer research community.

The progression of lung cancer is determined by a combination of environmental pollutants, genetic mutations, and tumour microenvironmental responses [40, 41, 42]. Therefore, this method can also be applied to a variety of inducible NSCLC and SCLC GEM models, representing common mutations found in human disease including KP or KL mice and alike [18, 43], by injecting adeno‐ or lentiviral Cre or FlpO recombinases, thus activating driver mutations specifically in lung epithelial cells. Of note, CRISPR‐Cas9 genome editing could also potentially be applied here. A transgenic mouse harbouring a *Cas9* allele was used and sgRNAs for a gene of interest injected orthotopically [44], thereby introducing additional mutations of interest specifically in the lung epithelial cells. This model also allows for the use of chemical carcinogens, or inflammatory agents, particularly relevant in early tumourigenesis research, such as urethane, that generates predominantly Kras‐driven lung ADC, mimicking early events of many human lung cancers [45]. Another application of this method would be to introduce pollutant particles (PM2.5) [42], tobacco, or air microplastics, thereby allowing the investigation of primary lung tumour initiation [46].

We also described here methods for *in vivo* longitudinal imaging allowing tracking of tumour growth and metastasis development using both cell lines and GEM models. Importantly, SPECT/CT has proved to be a highly sensitive methodology for tracking metastatic dissemination of cell lines, able to detect even small extra‐thoracic metastasis, while ultra‐focus thoracic CT is able to efficiently track development of primary tumour in both adenoviral and cell‐based unifocal models. This is especially relevant when assessing longitudinal responses to treatment.

This method also allows for specific targeted therapies to be used that require an identifiable single primary tumour, such as targeted radiation [39]. Additionally, it introduces the possibility of applying stereotactic radio‐ablation of the primary tumour to ‘mimic’ surgical resection of early‐stage tumours, combined with other therapeutic regimes, such as neoadjuvant chemo‐ or immunotherapy, thereby enabling further therapeutic strategies to be investigated.

### Limitations

One of the limitations of this methodology is the potential leakage of cells or adenovirus into the pleural fluid at the time of injection. We have addressed this by reducing the injection volumes (to 10 μl) and increasing the viscosity of the Matrigel carrier (using high‐concentration Matrigel). This refined approach resulted in reduced secondary tumour size but not incidence of secondary mediastinal and right lung lobe tumours, while the primary tumours in the left lung lobe reached greater volumes. Importantly, extra‐thoracic metastases were still found in most of the mice injected with LLCs. It is not possible to fully exclude that, in the case of tumours that reach large volumes, that cells may disseminate through pleural fluid, referred to as M1a category, in human lung cancer [47].

A further refinement of this technique could be to inject much smaller volumes (1–2 μl), which can be achieved best by stereotactic or ultrasound‐guided injection of tumour cells/adenovirus. The stereotactic method of implantation is routinely used for the implantation of cells in mouse brain [48, 49], as it enables accurate targeting deep within the tissue. Stereotactic injection into the lung allows greater precision during the injection compared with freehand injection and limits the injected volumes (supplementary material, Figure S8). It was previously shown that stereotactic injection of very low LLC cell numbers (500 cells) in 2–5 μl leads only to locally invasive disease, with no long‐distance metastasis observed up to 3 weeks after injection. When testing human cell lines in immunocompromised mice, injection of 10,000 H460 cells was found to lead to contralateral lung lobe and liver metastasis at 6 weeks after injection, but not earlier [50]. However, this stereotactic technique increases the surgery and anaesthesia time needed per mouse, making synchronous large experimental cohorts difficult to manage by a single user. Ultrasound‐guided lung injections would potentially omit the necessity to perform surgery; however, highly refringent bone tissue in ribs could potentially make this approach technically challenging.

## Conclusions

In summary, we presented here a detailed and refined protocol for the generation of unifocal lung cancer models with comparative analysis of disease progression, SPECT/CT based *in vivo* imaging methodology, and characterisation of immune and ECM content. The data presented here provide a guide to the differences in the biology of the various models developed. Importantly, the direct comparison of each model with human clinical pathological features has generated a useful ‘go‐to’ resource for the lung cancer research community for future studies (supplementary material, Tables S3, S4).

## Author contributions statement

A‐RP designed and performed the experiments. DM provided human lung sections and performed histopathological analysis. AC‐K performed LLC experiments. LR wrote part of the paper. RD took photos of the procedure. SA and ESahai performed the TWOMBLI analysis. AHardas performed histopathological analysis of metastases. YK assisted with experiments and writing of the paper. BW performed *in vivo* procedures and BW, JS, JC, JF and JK assisted with *in vivo* imaging. EM performed WGS analysis. JH performed the LLC subcutaneous *in vivo* experiments. EStern and OW developed the stereotactic mode of intralobular injection. GOF developed and CL characterised the mNIS reporter cell lines. A‐RP and KMH‐D conceived the project and wrote the paper.

## Supporting information


Supplementary materials and methods
**Figure S1.** Generation of *in vivo* traceable mouse lung cancer cell lines
**Figure S2.** Left lung intralobular surgical injection procedure
**Figure S3.** Progression of left lung lobe primary tumour growth and spread in unifocal lung intralobular models (extension to main Figure 1)
**Figure S4.** *In vivo* SPECT/CT longitudinal imaging to track growth of left lung lobe primary KP cell tumour (extension to main Figure 2)
**Figure S5.** Evidence of unifocal primary tumour in adenoviral‐Cre luciferase (30 μl in standard‐concentration Matrigel) injected into KP mice
**Figure S6.** Unifocal lung tumours induced by LLC cells injected (3,300 cells in 10 μl with standard‐concentration Matrigel) into C57Bl6 mice
**Figure S7.** Characterisation of lymphocytic and myeloid immune cell infiltration and extracellular matrix pattern of normal and tumour tissue in subcutaneous models for cell lines and multifocal adenoviral‐induced in KP mice
**Figure S8.** Left lung lobe intralobular stereotactic injection procedure
**Table S1.** Surgical troubleshooting
**Table S2.** Parameters used for TWOMBLI analysis
**Table S3.** Summary table of mutations identified in cell lines used and their relevance in NSCLC
**Table S4.** Summary table of features of lung cancer unifocal models presented in this study

**Movie S1.** Representative SPECT/CT maximum‐intensity projection movie at 21 days after LLC mNIS cell injection (10‐μl injection volumes in high‐concentration Matrigel) into left lung lobe (extension to Figure 4)

**Movie S2.** Representative SPECT/CT maximum‐intensity projection movie at 35 days after LLC mNIS cell injection (10‐μl injection volumes in high‐concentration Matrigel) into left lung lobe (extension to Figure 4)

## Data Availability

The data that support the findings of this study are openly available in Bioproject at https://www.ncbi.nlm.nih.gov/bioproject/PRJNA1220686, reference number 1220686.
