# Supplementary material for: Development and characterisation of improved unifocal primary mouse lung cancer models with metastatic potential
Source: J Pathol. 2025 Jun 18;266(4-5):405–20. doi: 10.1002/path.6435 (PMC12256383; doi:10.1002/path.6435)
Supplement: Supplementary file 1 — Supplementary materials and methods Figure S1. Generation of in vivo traceable mouse lung cancer cell lines Figure S2. Left lung intralobular surgical injection procedure Figure S3. Progression of left lung lobe primary tumour growth and spread in unifocal lung intralobular models (extension to main Figure 1) Figure S4. In vivo SPECT/CT longitudinal imaging to track growth of left lung lobe primary KP cell tumour (extension to main Figure 2) Figure S5. Evidence of unifocal primary tumour in adenoviral‐Cre luciferase (30 μl in standard‐concentration Matrigel) injected into KP mice Figure S6. Unifocal lung tumours induced by LLC cells injected (3,300 cells in 10 μl with standard‐concentration Matrigel) into C57Bl6 mice Figure S7. Characterisation of lymphocytic and myeloid immune cell infiltration and extracellular matrix pattern of normal and tumour tissue in subcutaneous models for cell lines and multifocal adenoviral‐induced in KP mice Figure S8. Left lung lobe intralobular stereotactic injection procedure Table S1. Surgical troubleshooting Table S2. Parameters used for TWOMBLI analysis Table S3. Summary table of mutations identified in cell lines used and their relevance in NSCLC Table S4. Summary table of features of lung cancer unifocal models presented in this study [file PATH-266-405-s001.docx]

**Development and characterisation of improved unifocal primary mouse lung cancer models with metastatic potential**

A-R Pedrosa *et al.* *J Pathol* <https://doi.org/10.1002/path.6435>

**Supplementary materials and methods**

**Supplementary Figures S1–S8**

**Supplementary Tables S1–S4**

Reference numbers refer to the main text list.

**Supplementary materials and methods**

**Materials**

***Reagents***

- DMEM with 4.5% glucose (Catalogue No.: 41966029, Gibco, Thermo Fisher Scientific, Altrincham, Cheshire, UK)
- Penicillin-streptomycin (Gibco, Catalogue No.: 15140122)
- Heat Inactivated Fetal Bovine Serum (HI FBS) (Gibco, Catalogue No.: 10438026)
- Matrigel Matrix (with growth factors: Catalogue No.: 356234 for 30-µl injections (standard concentration); Catalogue No.: 354263 for 10-µl injections (high concentration), aiming for a 9–9.5 mg/ml final Matrigel concentration, Corning, Ewloe, Flintshire, UK)
- 0.25% Trypsin-EDTA (Gibco, Catalogue No.: 25200072)
- Dulbecco’s PBS (Catalogue No.: D8537, Sigma-Aldrich, Merck, Feltham, Middlesex, UK)
- Calcium chloride (CaCl_2_) (Catalogue No.: 19273, Acros Organics, Thermo Fisher Scientific)
- Minimal Essential Medium (MEM) (Gibco, Catalogue No.: 21090-022)
- Rely+On Virkon tablets (LanXess solutions, UK); dissolve 1×5 g Virkon tablet per 500 ml of water to prepare a 1% working solution.
- L-Glutamine solution (Catalogue No.: G7513, Sigma-Aldrich)
- Isoflurane, 100% (w/w) (Vm 42058/4195, Zoetis, Letherhead, Surrey, UK)
- Ophthalmic ointment (AaCarb Carbomer 0.2% eye gel, TriON Pharma, Coventry, West Midlands, UK)
- Water for injections (100% v/v solvent for parental use, Norbrook Laboratories, Market Harborough, Leicestershire, Northern Ireland, UK)
- Formalin solution, neutral buffered, 10% (HT501128, Sigma-Aldrich)
- Alcohol (methylated spirit industrial, 74 O.P.), 99% (v/v) (Catalogue No.: M/4450/17, Thermo Fisher Scientific).

***Prescription drugs***

- Buprenorphine (0.1 mg/kg)-Vetergesic (Ceva Animal Health Ltd, Wooburn Green, High Wycombe, UK)
- Xylazine (10 mg/kg), Rompun (Bayer, Reading, Berkshire, UK)
- Ketamine (100 mg/kg), Narketan (Vetoquinol Ltd, Steadings Barn, Towcester, UK)

***Surgical equipment***

- Lab animal anaesthetic setup (including Isofluorane pour fill vaporisers-Tec3; induction chamber; breather circuit; flowmeters, quick release 5% vaporisers; scavenging system and Clini-Ox 5L Oxygen concentrator, CliniPath equipment Ltd, Hull, Kingston upon Hull, UK)
- Mouse recovery rack (CliniPath equipment Ltd)
- Autoclaved nitrile gloves (Catalogue No.: 93833070 Microflex, StarLab Ltd, Milton Keys, Buckinghamshire, UK)
- Surgical instruments (Fine Science Tools, Heildelberg, Germany) including standard pattern forceps straight (11000-20), fine scissors straight (91460-11), and blunt scissors (such as Metzenbaum scissors blunt 91417-18)
- Autoclaved surgical drape (3M Science applied to life, Bracknell, Berkshire, UK)
- Autoclaved surgical gown (3M Science applied to life)
- Press’n Seal Sealing wrap (Glad, Amazon, Shoreditch, London, UK)
- Autoclaved cotton tip applicator
- For 30-µl injections: 27 G (3/4 in.) needle (BD Microlance 3 302200, BD, Wokingham, Berkshire, UK) and 1-ml syringe (HENKE-JECT Tuberkulin 5010-200V0, Avantor by VWR, Lutterworth, Leicestershire, UK)
- For 10-µl injections: BD Microfine U-100 insulin 0.5-ml syringe with 29 G needle (BD Microfine 324892) or 100 µl Hamylton Syringe (Catalogue No.: 80601, Merck)
- Optional: Fine bore polythene tubing (0.38 mm ID and 1.09 mm OD) (Catalogue No.:800/100/120, Portex, smiths medical, London, UK). This tube can be used as an alternative to marking the needle to serve as a needle stopper. Cut 7-mm sections and insert into needle, so that the free needle has 5 mm.
- Veterinary kit (Catalogue No.: IN015, Vet-Tech Solutions, Congleton, Cheshire, UK) including: Clay Adams 9-mm wound clips (Catalogue No.: 427631); Clay Adams wound clip remover (SLS; INS4956 D2-280), and clippers (DMS Veterinary, Blacknest, Alton, UK; GT421G).

***Additional surgical equipment (for surgical closing using sutures)***

- Coated Vicryl Rapide suture (Catalogue No.: W9913, Johnson & Johnson MedTech/Veterinary, Wokingham, Berkshire, UK)
- Microlance Needle (0.5 × 25 mm) (BD, Catalogue No.: 300400)
- Stainless steel S&T Suture Tying Forceps (Fine Science Tools, Catalogue No.: 00272-13)
- Crile-Wood Needle Holders (Fine Science Tools, Catalogue No.: 12003-15)

***Additional surgical equipment (for intratracheal administration of adenovirus)***

- Exel Safelet IV catheters (22 gauge, 1 in., Catalogue No.: 14-841-20, Thermo Fisher Scientific)
- Intubation platform (Steve Boukedes, labinventions@gmail.com)
- Fiber-Lite Illuminator (Model 3100-1, Dolan-Jenner Industries, Setra, MA, USA)

**Reagent preparation**

- **Medium for cell growth**: Modify DMEM by adding 1% (v/v) of Pen Strep and 10% (v/v) of FBS.
- **Preparation of analgesic (buprenorphine 0.1 mg/kg)**: Prepare a 1/10 working solution in injectable water. Each mouse should receive 100 µl of working solution into the scruff prior to surgery.
- **Preparation of injectable anaesthetic**: Dilute xylazine (0.8/10) and ketamine (2/10) in injectable water. Each mouse should receive 100–150 µl i.p. (according to body weight).
- Prepare a solution of ethanol 70% by diluting alcohol 99% in dH_2_0.

**Methods**

**Animals**

Animals were housed in groups of four to six mice per individually ventilated cage in a 12-h light/ dark cycle (06:30–18:30 light; 18:30–06:30 dark), with controlled temperature (21±1 °C) and relative humidity (40–60%). The cages contained a 1- to 1.5-cm layer of animal bedding, with environmental enrichment, including a card-board box tunnel and crinkled paper nesting material. The animals had access to food and water *ad libitum*.

**Tumour cell culture and preparation**

Cells were grown at 37 °C and 5% CO_2_ using standard cell line-specific procedures. Cells were washed with PBS and detached with trypsin at 37 °C. Trypsin activity was quenched by adding an equal volume of medium, and the cell suspension was centrifuged at 12,00 rpm for 3 min to pellet the cells as the LLC cell line has both adherent and floating cell populations. PBS was used to detach adherent cells from tissue culture plates. (Use both floating and adherent populations mixed for cell injections.) The cell pellet was washed twice in PBS and pelleted by centrifugation. After the second wash, the pellet was resuspended in the appropriate volume of PBS and cells counted using a Fast-read 102 cell counting slide. After pelleting down, cells were resuspended in an appropriate amount of PBS as per calculations.

**Whole-genome sequencing**

Genomic DNA was isolated following the manufacturer’s protocol (DNAeasy Kit, Qiagen, Manchester, UK; Catalogue No.: 69504) and sequenced at Novogene (Milton, Cambridge, UK). Whole-genome sequencing was performed on the NovaSeq XPlus, with 150-bp, paired-end reads, yielding ~67 million sequencing reads per sample, paired-end. After an initial quality check using fastqc version 0.11.5, reads were quality trimmed using trimgalore version 0.6.5 and aligned to the human reference genome GRCm38 (m10) using bwa version 0.7.17. Picard version 2.25.7 was used to mark read duplicates and assess insert size distributions. Base quality score recalibration was performed on known sites of variation using gatk version 4.2.1.0 and 00-All.vcf.gz and mgp.v5.indels.pass.chr.sort.vcf.gz, downloaded from NCBI and Sanger mgP. Variant calling was performed using gatk-4.2.1.0 mutect2 on tumour-only mode. VCF files were further filtered using gatk FilterMutectCalls followed by refgene and cytoband annotation in annovar. Further filtering was subsequently applied to obtain variants with sequencing depth DP > = 2 and AF > 0.1. Non-synonymous mutations were used for graphical representations. CNV analysis was performed using R packages QDNAseq and Rascal [51]. Whole-genome sequencing data were deposited in the SRA database, accession number PRJNA1220686.

**Sodium iodide symporter (mNIS) reporter**

All *in vivo* traceable cell lines (LLC, CMT, and KP) were genetically engineered to express a stable murine NIS fused C-terminally to enhanced green fluorescent protein carrying the monomerising A206K mutation (mNIS-GFP). This approach was previously shown to provide cancer cell tracking with excellent sensitivity on the whole-body level while being quantitative and non-invasive [15–17]. In brief, lung cancer cell lines were transduced with lentiviruses carrying the mNIS-GFP transgene under control of a spleen focus-forming promoter, purified (from parental non-transduced cancer cells) by fluorescence-activated cell sorting, and characterised using established methodology assessing reporter expression, subcellular localization, and function (by radiotracer Tc-99m-pertechnetate uptake). For more details on constructs and characterisation data, please see supplementary material, Figure S1C–E.

**Step-by-step thoracotomy protocol**

**∆ CRITICAL highlights special attention for the required step.**

**! CAUTION indicates steps where special caution will be required.**

1. One day prior to surgery, mice were shaved in the left thorax area.

**∆ CRITICAL** Shaving one day prior to surgery prevents shaved fur contaminating the wound and potentially causing infection.

1. Analgesia [Vetergesic (buprenorphine), 0.1 mg/kg] was administered subcutaneously (0.1 ml) in the scruff.
2. Mice were anaesthetised in the induction chamber (with 3–4% isoflurane rate) and ophthalmic lubricant applied to the eyes once the mouse was fully anaesthetised. The mouse was laid on its right side, so that the left side faced the operator (supplementary material, Figure S2A) with the muzzle inserted into the anaesthetic nose piece to maintain anaesthesia, using a reduced flow of isoflurane of 2–2.5%.
3. The mouse was covered with a sterile surgical drape (3M Science applied to life) or Press’n Seal Sealing wrap (Glad, Amazon) with a window exposing only the left thorax (supplementary material, Figure S2A).
4. The shaved area (surgical field) was sterilised with 70% ethanol/betadine/chlorhexidine.
5. The most posterior ribcage limit was identified, and then, using sterile sharp-ended scissors, a lateral transversal incision was made (5–10 mm in length) in the left thoracic wall, 5 mm posterior to the scapula (mid-point between scapula and ribcage limit) (supplementary material, Figure S2B).
6. Using a small set of blunt scissors, blunt dissection of the subcutaneous space was performed, removing and separating fat and muscle to visualise the pleural with thoracic ribs, intercoastal spaces, and left lung lobe underneath (supplementary material, Figure S2C–E).

**∆ CRITICAL** It is important to change the set of surgical instruments after cutting the skin surface and tissues under the skin. This will minimise the risk of introducing infection.

1. The syringe was loaded with the premarked needle, or with the needle stopper tube inserted, containing the cell suspension or viral suspension. The needle was applied to the syringe and bubbles removed by expelling liquid until no bubbles were present. The volume was adjusted to the exact loading volume to be injected. (supplementary material, Figure S2F).

**∆ CRITICAL** Prepare the injection depth by drawing a black line with a fine permanent marker 5 mm from the needle tip as a reference point for the depth at which the needle needs to be inserted (supplementary material, Figure S2F). Optional: Use the needle stopper tube (same width as the needle and 5 mm shorter than the needle length) inserted over the needle (fine bore polythene tubing, Portex/Smiths Medical, cut 7-mm sections and insert into needle). Ensure that the tubes containing cells or virus are mixed well prior to loading into the syringe, as the cells will have settled at the bottom of the tube and may be clumped together.

1. The needle was positioned, with the bevel up, between the fifth and sixth ribs at a 45° angle relative to the mouse body and pointing towards the scapula (supplementary material, Figure S2G).
2. With a precise and sharp movement, the needle was inserted to a depth of 5 mm (indicated by the guideline on the needle or by the tube stopper), and holding the needle in place, the cell suspension was injected into the left lobe.

**∆ CRITICAL** This sharp movement ensures that the needle will penetrate the very soft lung tissue and avoid injection into the thoracic cavity, just between the pleura and lung, rather than inside the left lung lobe. See supplementary material, Figure S2H, for a schematic guide.

1. A sterile cotton tip applicator was gently applied at the site of the injection, while simultaneously withdrawing the needle slowly. This stopped any bleeding and avoided the cell suspension from leaking out of the lung tissue (supplementary material, Figure S2I).

**! CAUTION** Immediately after injection, the respiration and heart rate of the mouse may increase significantly. However, both should stabilise within 2 min.

1. The two skin flaps were brought together using two pairs of rounded forceps and lifted gently to create a space between the skin and the organs underneath, and the wound was closed using wound clips or sutures (supplementary material, Figure S2J,K).

**! CAUTION** Avoid creating an air pocket when closing the two skin flaps.

1. The mouse was removed from the anaesthetic mask and laid on its left side in a clean cage under a heat lamp or heated pad to recover (supplementary material, Figure S2L).

**∆ CRITICAL** The mouse must be laid on its left side to prevent possible leakage of the Matrigel suspension from the left lobe into other lobes and pleural fluid.

See supplementary material, Figure S2M, for a schematic diagram of a unifocal tumour in the left lung and nomenclature of right lung lobes 1–4.

**Postoperative care monitoring**

Mice were monitored regularly until they were mobile and had recovered from the anaesthetic. The mouse cages were then returned to the IVC housing racks. Monitoring of the mice continued regularly, and 0.1 ml analgesic (Vetergesic, 0.1 mg/kg) was administered subcutaneously into the scruff of the mouse, 6–8 h after surgery. A further dose of analgesic was administered the following morning (24 h after surgery), and monitoring continued. It is recommended, according to Home Office rules, that mice be routinely monitored every day. Between 7 and 10 days after surgery, the wound clips were removed using a wound clip remover.

**Table S1. Surgical troubleshooting**

| **Step** | **Problem** | **Possible reason** | **Solution** |
| --- | --- | --- | --- |
| 6–7 | Bleeding in skin | Capillaries cut during  incision | Apply pressure with sterile cotton bud directly onto site of bleeding |
| 8–9 | Matrigel begins to solidify and blocks syringe | Matrigel not cooled properly | Make sure that the Matrigel cell suspension is always on ice during procedure |
| 10 | Damage to lung | Operator inadvertently inserts needle through left lung lobe. This could result in a haematoma, from which the mouse may not recover | Training on cadavers is important.  Make sure needle has mark on it to restrict depth to 5mm |
| 11 | Leakage of cells/Matrigel immediately after injection into left lung lobe | Operator removes needle too quickly from lung after injection | Remove needle slowly after injection of Matrigel cell and use cotton swab to prevent leakage from site |
| 12 | Possible trapping of air when closing wound | Lifting edges of wound too high | Remove wound clips and re-close without trapping air |
| 6–12 | Risk of infection | Aseptic technique not followed | Make sure all surgical instruments are sterilised, follow aseptic technique during surgery |
| 13 | Animal not recovering as expected after surgery is complete and does not breathe spontaneously | Haematoma after injection | Mouse will be culled |
| Post-recovery | Wound clips may come out and wound may re-open | Wound clips applied incorrectly | Anaesthetise mouse and re-apply clips |
| During development of tumours | Thoracic wall or subcut tumours develop on site of injection | Leakage of cell or viral suspension | Exclude mice from study  Make sure to apply cotton bud with a bit of pressure and before removing syringe  Wipe clean needle after loading syringe and before injection |

**SPECT/CT and respiratory gated lung CT equipment setup**

SPECT imaging was performed using HE-GP-M SPECT collimators with 1.4-mm pinholes and energy window set to record Tc-99m-pertechnetate radiation and full detector rotation over XY min. CT imaging was performed with the following parameters: scan angle = 360° stepping with 75 ms exposure at each frame, tube current of 0.21 mA, tube voltage of 50 kV. SPECT scans were reconstructed to a voxel size of 0.4 μm using an SROSEM algorithm with nine iterations and 128 subsets and a Gaussian filter of 0.5 mm full width half maximum. CT scans were reconstructed with a voxel size of 80 μm. SPECT and CT scans were co-registered and attenuation corrected using XY software. Region of interest analysis of the coregistered SPECT and CT images was performed using VivoQuant image analysis software (inviCRO LLC 2021patch1hf1). Primary tumour foci total MBq activity was calculated using the following formulas (calibration factor was measured for Tc99m using the same specifications as for the mouse scans):

$$CF= \frac{Expected activity \left( \mathrm{MBq} \right)\times Number of voxels}{\mathrm{Volume}\left( mm^{3} \right)\times Sum}$$

$$\text{Activity }\left( \text{MBq} \right)=$$

$$=\text{Mean × Calibration factor (MBq/m}\text{m}^{\text{3}}\text{) × Volume (m}\text{m}^{\text{3}}\text{)}$$

Gated CT imaging was performed with the following parameters: magnification = ultrafocus step and shoot; scan angle = 360° stepping with respiratory gated 20 ms exposure at each frame, tube current of 0.21 mA, tube voltage of 50 kV. The CT scan was reconstructed with a voxel size of 40 µm and gated for respiration.

**Tissue collection and histological analysis**

Mice were culled by cervical dislocation and lungs inflated with formalin. Lungs were removed and incubated overnight in formalin at room temperature. Formalin was then replaced with 70% ethanol and samples stored at 4 °C. The individual lung lobes were separated and positioned in the embedding cassette as shown in supplementary material, Figure S3. Lungs were embedded in paraffin and sectioned for H&E staining and other markers as appropriate. Details of H&E, CD3, CD11, GFP, and Picrosirius Red Stain are below. Slides were scanned using a Pannoramic 250 High Throughput Scanner (3DHISTECH, Budapest, Hungary). Analysis of left lung tumour focus area was undertaken using CaseViewer software (3DHISTECH). Analysis of other immunostaining was performed using QuPath image analysis software (https://qupath.github.io/). Analysis of a broad range of metrics from the ECM in the primary tumour was perfomed using TWOMBLI [25] (The Workflow Of Matrix BioLogy Informatics) image analysis (as described in “ECM pattern analysis using TWOMBLI” below).

**Equipment for tissue preparation and immunohistochemistry**

- Fast-read 102 (10 chamber) slide for cell counting (Catalogue No.: 630-1893, Avantor, Lutterworth, Leicestershire, UK)
- Centrifuge 5810 for 15/50 ml tubes (Catalogue No.: 5810000060 Eppendorf, Stevenage, Hertfordshire, UK)
- HistoCore Arcadia H (paraffin embedding) used with a cooling system HistoCore Arcadia C (Leica Biosystems, Linford Wood, Milton Keynes, UK)
- Microtomes HistoCore Autocut RM2255 or RM2235 (Leica Biosystems)
- Ventana Discovery Ultra machine (Roche Diagnostics Ltd., Burgess Hill, West Sussex, UK)
- Leica Autostainer XL (version 2.01) (Leica Biosystems, Catalogue No.: ST5010)
- Contura Trimmer (Wella Professionals, Wimbledon, London, UK)

**Reagents for tissue preparation**

- Paraffin: Surgipath Paraplast Bulk (Leica Biosystems, Catalogue No.: 39602012)

**Reagents for H&E staining**

- Xylene, 97% (Catalogue No.: X/0200/17, Thermo Fisher Scientific)
- Alcohol (methylated spirit industrial, 74 O.P.), 99% (v/v) (Catalogue No.: M/4450/17, Thermo Fisher Scientific)
- Haematoxylin Gill 3 (Leica Biosystems, Catalogue No.: 3801542)
- Surgipath Eosin (Leica Biosystems, Catalogue No.: 3801601E)
- 1% Acid Alcohol (Leica Biosystems, Catalogue No.: 3803651E)

**Reagents for CD11b, CD3 and GFP staining**

- DISCOVERY wash solution (Catalogue No.: 950-510 Ventana Medical Systems, Roche Diagnostics Ltd.)
- Reaction buffer 10X (Ventana Medical Systems, Catalogue No.: 950-300)
- LCS (predilute) (Ventana Madical Systems, Catalogue No.: 650-010)
- Cell conditioning solution (CC1) (Ventana Medical Systems, Catalogue No.: 950-124)
- DISCOVERY antibody diluent (Ventana Medical Systems, Catalogue No.: 760-108)
- DISCOVERY OmniMap anti-Rb HRP (Ventana Medical Systems, Catalogue No.: 760-4311)
- DISCOVERY ChromoMap DAB kit (Ventana Medical Systems, Catalogue No.: 760-159)
- Haematoxylin 2 (Ventana Medical Systems, Catalogue No.: 790-2208)
- Bluing reagent (Ventana Medical Systems, Catalogue No.: 760-2037)
- Rabbit monoclonal anti-CD11b antibody, 1:4000 (Catalogue No.: ab133357, Abcam, Cambridge, Cambridgeshire, UK)
- Rat monoclonal anti-CD3 antibody, 1:300 (Abcam, Catalogue No.: ab11089)
- **Rabbit GFP Polyclonal antibody, 1:500 (**Thermo Fisher Scientific**, Catalogue No.: A-11122)**
- Rabbit anti-rat IgG antibody (Catalogue No.: AI-4001, Vector Laboratories- 2B Scientific, Kirtlington, Oxfordshire, UK)
- Omnimap anti-rabbit HRP (Roche, Catalogue No.: 760-4311)

**Reagents for Picrosirius Red staining**

- Picrosirius Red stain (Direct red 80) (Sigma-Aldrich, Catalogue No.: 365548)
- Picric acid (saturated aqueous solution) (Catalogue No.: 640765 Generon, Slough, Berkshire, UK)
- Acetic acid, 99+%, glacial (Thermo Fisher Scientific, Catalogue No.: 11317558)
- Weigert’s Haematoxylin Solution A (Catalogue No.: RRSP72-D Atom Scientific Ltd, Hyde, Greater Manchester, UK)
- Weigert’s Haematoxylin Solution B (Catalogue No.: RRSP73-D, Atom Scientific Ltd)

**Reagent preparation**

Picrosirius Red mixture: add 0.5 g of Picrosirius Red stain to 500 ml of aqueous solution of picric acid and mix gently. Picrosirius Red mixture can be stored for up to 3 years and reused multiple times.

0.5% Acidified water: add 5 ml acetic acid to 1 l distilled or tap water. Mix well.

Weigert’s Haematoxylin solution: add 25 ml of Weigert’s Haematoxylin Solution A and 25 ml Weigert’s Haematoxylin Solution B into a clean container. Mix well.

**H&E staining protocol**

The Autostainer XL machine stains the slides automatically. The machine has separate containers for the reagents used for staining (stations 1–18) and washing (wash 1–5). There are also ‘load’ and ‘exit’ stations.

1. Place paraffin-embedded slides in staining basket in loading station and press start.
2. The slides will move from station to station as per the following programme:

| Station 1 | 95% xylene for 3 min | Step 1 – deparaffinisation |
| --- | --- | --- |
| Station 2 | 95% xylene for 3 min |  |
| Station 3 | 95% xylene for 3 min |  |
| Station 4 | 100% alcohol for 3 min | Step 2 – rehydration |
| Station 5 | 100% alcohol for 3 min |  |
| Station 6 | 90% alcohol for 3 min |  |
| Station 7 | 70% alcohol for 3 min |  |
| Wash 1 | Running tap water for 2 min | |
| Station 8 | Haematoxylin solution for 3.5 min | Step 3 – staining |
| Wash 2 | Running tap water for 4 min | |
| Station 9 | 1% Acid alcohol for 20 s | Step 4 – differentiation |
| Wash 3 | Wash with running tap water for 3 min | |
| Station 10 | Eosin solution for 6 min | Step 5 – staining |
| Wash 4 | Running tap water for 1:30 min | |
| Station 11 | 70% alcohol for 1.5 min | Step 6 – dehydration |
| Station 12 | 90% alcohol for 2 min |  |
| Station 13 | 100% alcohol for 2 min |  |
| Station 14 | 100% alcohol for 3 min |  |
| Station 15 | 95% xylene for 2 min |  |
| Station 16 | 95% xylene for 2 min |  |

At the end of the programme, the slides are placed in the exit station.

**CD11b and GFP immunohistochemistry protocol**

Use Ventana Discovery ULTRA machine for automated CD11b and GFP staining. It is recommended to use 4-µm-thick paraffin-embedded tissue sections.

1. Before starting, choose the appropriate reagents (as described above) and install the reagent tray.
2. Place the slides in the independent slide drawers and load the protocol into the system.
3. Run the protocol as outlined in table below.
4. After staining, take the slides out and wash them in tap water with detergent.
5. Dehydrate the slides in increasing concentrations of ethanol and xylene.
6. Mount and coverslip the slides.

| **Step** | **Conditions** |
| --- | --- |
| Deparafinisation in Discovery wash solution | 75$℃$, 3×8 min cycles |
| Cell conditioning in Tris-EDTA-based conditioning solution (CC1) | 95 $℃$, 32 min |
| Blocking in inhibitor (cm) | 37 $℃$, 4 min |
| Rabbit CD11b/ GFP primary antibody incubation (100 µl) | 60 min |
| Omni-Map HRP secondary antibody incubation (one drop) | 16 min |
| DAB cm (one drop) and H_2_O_2_cm (one drop) incubation | 8 min |
| Copper cm (one drop) incubation | 5 min |
| Counterstain with haematoxylin 2 | 8 min |
| Post-counterstain with bluing agent | 8 min |

**CD3 immunohistochemistry protocol**

The Ventana Discovery ULTRA machine is used for automated CD3 staining. It is recommended that paraffin-embedded tissue sections 4 µm thick be used.

1. Before starting, prepare appropriate reagents (as described above) and install reagent tray.
2. Place slides in independent slide drawers and load protocol into system.
3. The protocol steps are detailed in table below.
4. After staining, remove slides and wash them in tap water with detergent.
5. Dehydrate slides in increasing concentrations of ethanol and xylene.
6. Mount and coverslip slides.

| **Step** | **Conditions** |
| --- | --- |
| Deparafinisation in Discovery wash solution | 75 $℃$, 3×8 min cycles |
| Cell conditioning in Tris-EDTA-based conditioning solution (CC1) | 95 $℃$, 32 min |
| Blocking in inhibitor (cm) | 37 $℃$, 4 min |
| Rat CD3 1$^{\circ}$ antibody incubation (100 µl) | 60 min |
| Rabbit anti-rat 2° antibody incubation (one drop) | 42 $℃$, 20 min |
| Omni-Map anti-rabbit HRP (one drop) | 16 min |
| DAB cm (one drop) and H_2_O_2_cm (one drop) incubation | 8 min |
| Copper cm (one drop) incubation | 5 min |
| Counterstain with haematoxylin 2 | 8 min |
| Post-counterstain with bluing agent | 8 min |

**Picro-sirius red staining protocol**

Ideally, tissue sections should be 4–5 µm thick.

1. Dewax tissue sections in three changes of 95% xylene (2 min each).
2. Rehydrate sections in decreasing concentrations of alcohol (2×2 min incubations in 100% ethanol, 1 min incubation in 90% ethanol, and 1 min incubation in 70% ethanol).
3. Immerse slides in working Weigert’s Haematoxylin solution for 5 min.
4. Wash slides for 2 min with running tap water.
5. Place slides in Picrosirius Red mixture for 1 h.
6. Wash slides in two changes of 0.5% acidified water.
7. Dry slides by shaking vigorously and dehydrate them by incubating in three changes of 100% ethanol (2 min each).
8. Mount slides as desired.

**QuPath analysis of Cd3 and Cd11b positive cells**

From each mouse primary tumour, three to five tumour edge/boundary regions of interest (ROIs) and three to five tumour core ROIs were drawn, and positive cell detection analysis was used to measure the percentage of positive Cd3 and Cd11b (used cell_DAB max signal for identifying positive cells) out of the total of cells present in each ROI. The full script is available upon request to the corresponding author.

**ECM pattern analysis using TWOMBLI [25]** **(FIJI macro for quantifying pattern in extracellular matrix)**

From each sample’s Picrosirius Red stained sections, three tumour and three adjacent normal tissue regions (300 × 300 µm each) were chosen and exported as .tif images using QuPath-0.4.3 software (https://qupath.github.io/). Colour deconvolution was performed to isolate the ECM channel (red) using ImageJ 1.53t software (https://imagej.net/ij/) with the following RGB values ([r1]=0.069 [g1]=0.730 [b1]=0.6797 [r2]=0.0463 [g2]=0.350 [b2]=0.9355 [r3]=0.1506 [g3]=0.5820 [b3]=0.799). These ECM images were then processed using the TWOMBLI pipeline [25] after identifying the analysis parameters (Table S2) on an example set of images derived from the main set. PCA was then performed in Graphpad Prism 9 using all the output parameters from TWOMBLI as variables to visualise the sample clusters.

**Table S2.** **Parameters used for TWOMBLI analysis**

**
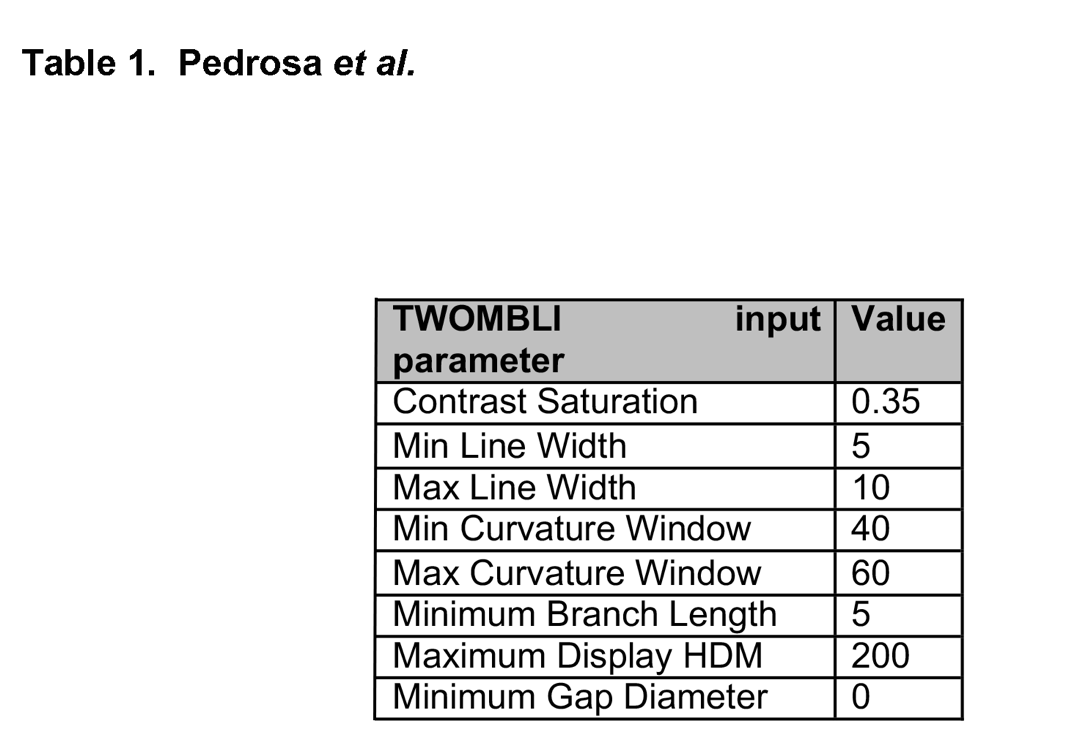
**

**
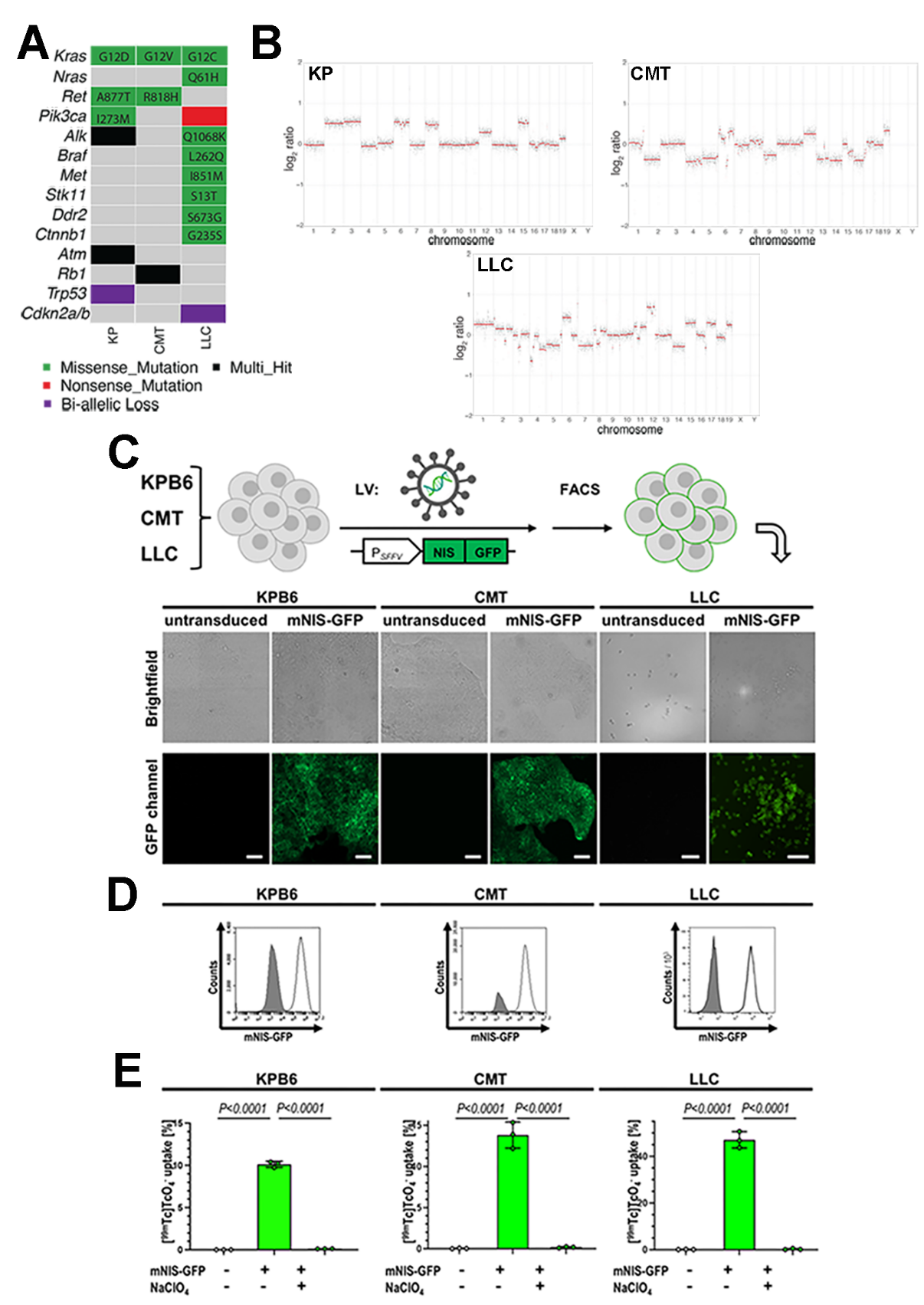
**

**Figure S1. Generation of *in vivo* traceable mouse lung cancer cell lines.** (A) Oncoplot illustrating non-synonymus mutations and bi-allelic deletions in oncogenic drivers in the three cell lines. (B) Copy number profiles of the three cell lines. (C) Cartoon depicting cell engineering workflow. The pLNT/SFFV mNIS-GFP construct was generated in an analogous way as described for the human NIS-GFP variant described by Volpe *et al* [15]. Lentivirus particles were produced in HEK293T cells with DMEM as base medium using methodology described by these authors. Three million of each indicated lung cancer cell line received freshly produced lentivirus particles (1+1 diluted with corresponding fresh fully supplemented growth medium), and cells were transduced for 72 h before receiving fresh growth medium and being expanded to 90% confluency in T75 flasks. Cells were fluorescence-activated cell sorting (FACS) sorted and grown to stability before cell line characterisation commenced. Brightfield and fluorescence microscopy of non-transduced (control) and transduced cell lines prior to cell sorting. In mNIS-GFP-expressing cells, fluorescence is predominantly visible in cell membranes, which is in line with expectations for the mNIS-GFP reporter that requires plasma membrane localisation for correct function. (D) Flow cytometry reveals purity of FACS-sorted cell lines (measurements were taken three passages after cell sorting). Overlays are shown with grey-shaded curves indicating non-transduced control cells and open curves indicating purified mNIS-GFP-expressing lines. (E) mNIS-GFP reporter function was determined by uptake of the radiotracer [^99m^Tc]TcO_4_^−^ as previously described [14–17]. mNIS-GFP-expressing cell lines showed radiotracer uptake, while non-transduced cells did not. Importantly, radiotracer uptake in mNIS-GFP-expressing cells was sensitive to the competitive substrate perchlorate (used at 12.5 μM), demonstrating that radiotracer uptake was specific to mNIS. **Schematics were created in BioRender.comSchematics were created in BioRender.com.**

**
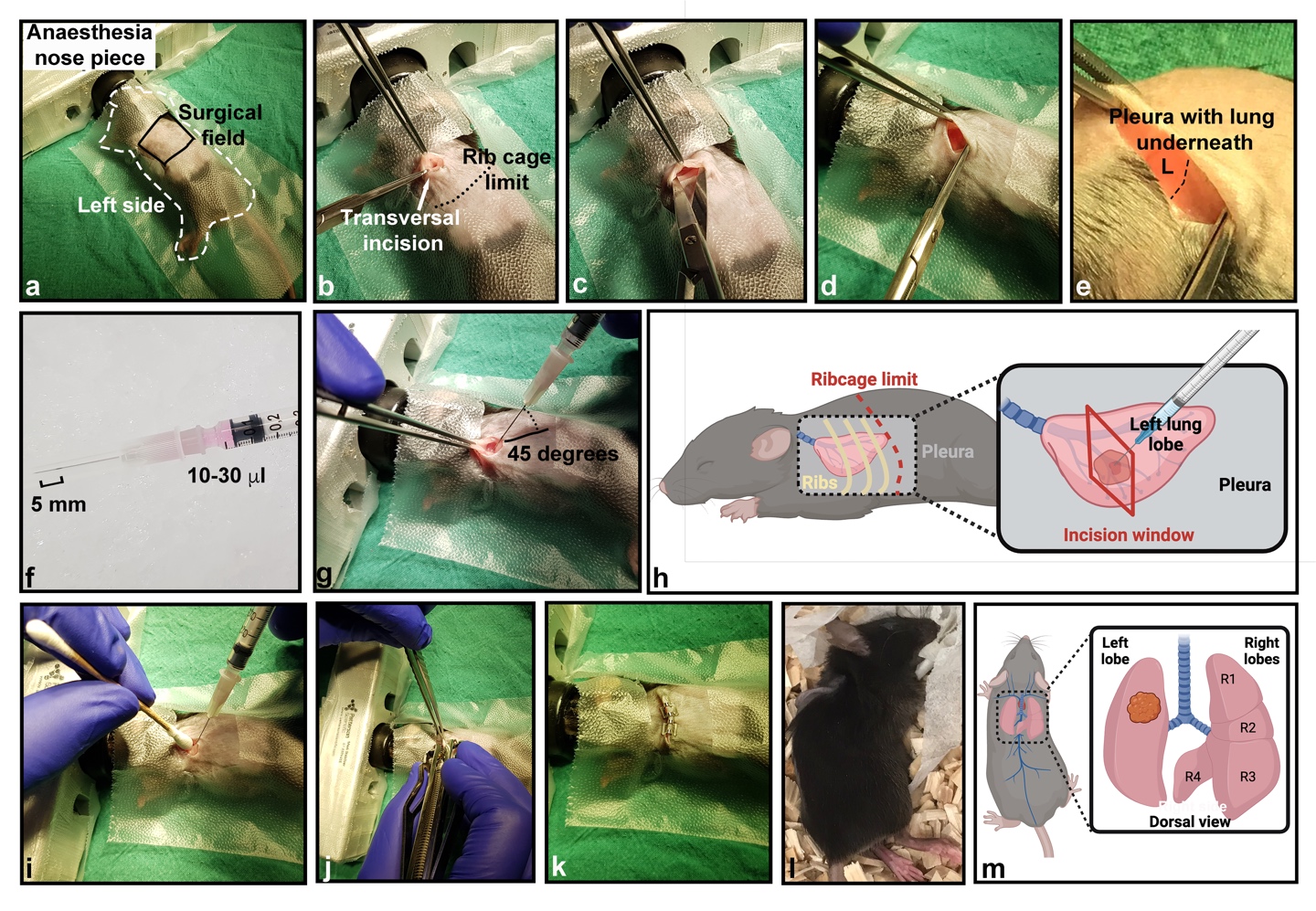
**

**Figure S2.** **Left lung intralobular surgical injection procedure.** Representative images of left lung lobe surgical injection of tumour cells in C57Bl6 mice**.** (A) Surgical field: mouse lying on its right side, with shaved left side, denoted by *white dashed line*. Exposed surgical field is denoted by continuous *black square*. (B) Transverse incision site: Dashed *back line* denotes rib cage limit and *white arrow* the vertical incision mid-point between the rib cage limit and scapula. (C) Blunt dissection: using tweezers and point blunt scissors debridement of the subcutaneous tissue to expose intact thoracic cavity underneath. (D and E) Exposure of ribcage and pleural cavity. The lung (L) is seen through the translucent pleura beneath as a whitish structure. (F) Injection depth marking of needle: syringe on ice preloaded with 30 µl cell suspension in Matrigel and needle marked for 5 mm injection depth. (G) Injection angle: positioning of syringe with needle bevel up, 45° relative to mouse body and pointing towards scapula for injection between fifth and sixth ribs. (H) Schematic representation of left lung lobe in surgical field and details of injection site. (I) Pressure application: Before removing the needle from the point of injection, a cotton bud tip is applied to apply slight pressure and avoid cell leakage into thoracic wall or cavity. (J and K) Closing the wound: Both sides of the incision are brought together using two pairs of tweezers and two clips are applied to close the wound. (L) Mouse positioned lying on its left side to recover in a clean cage on a heated surface. (M) Schematic representation of dorsal view of anatomical position of the five lung lobes and the location of the primary tumour that develops with this procedure. Schematics were created in BioRender. Pedrosa, A. (2025) https://BioRender.com/6m9rxd3.

**
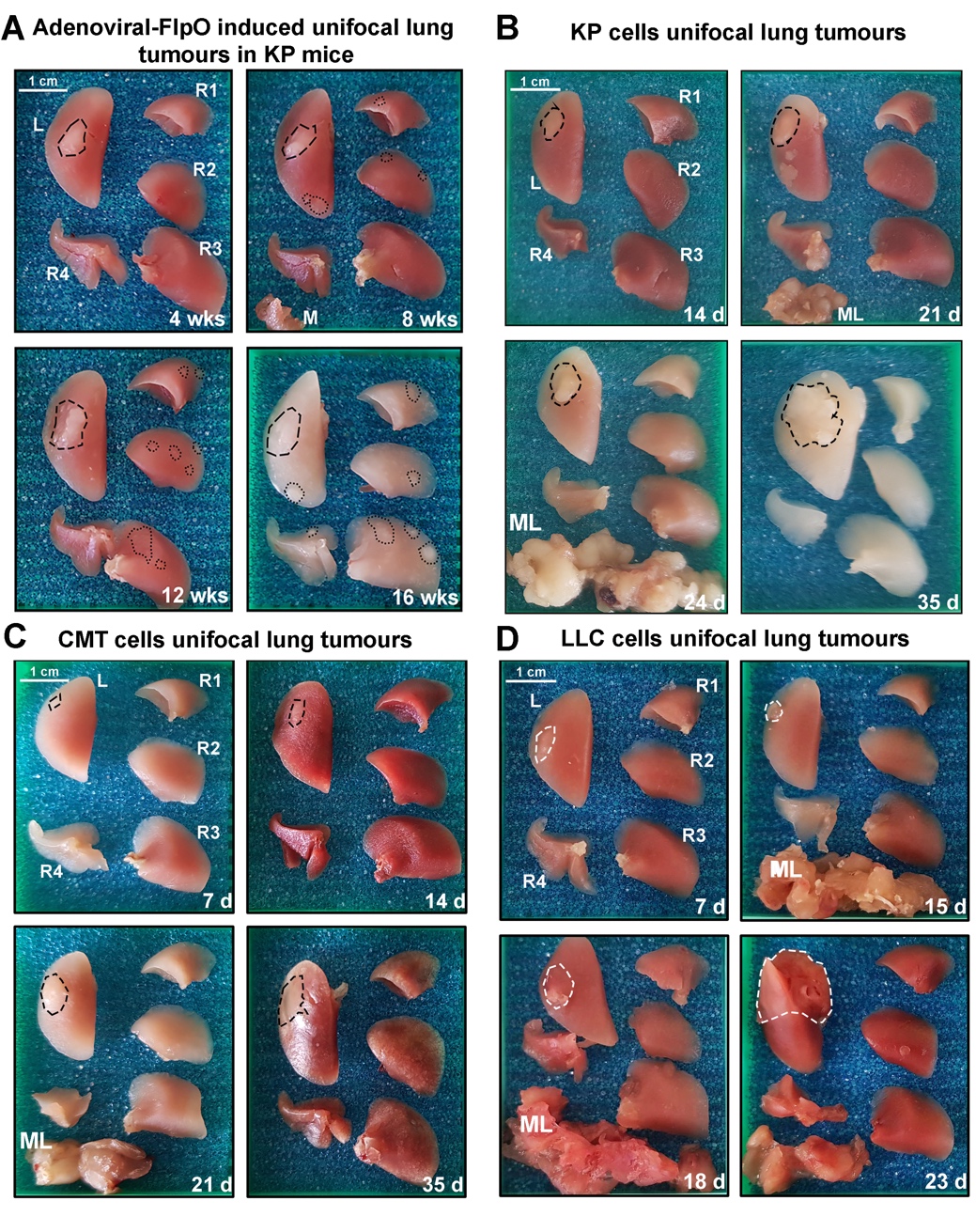
**

**Figure S3. Progression of left lung lobe primary tumour growth and spread in unifocal lung intralobular models** **(extension to main Figure 1).** Representative images of the five mouse lung lobes together with mediastinal structures (with tumours) at the bottom left, at progressive time points after adenoviral-FlpO administered in KP mice, or KP, CMT, and LLC (30 μl) cell injections into left lung lobe. Dashed lines highlight primary tumour foci in left lung lobe (L). *R1*, *R2*, *R3*, *R4*, right lung lobe numbers and *ML*, mediastinal lymph node tumours. (A) Adenoviral-FlpO-induced unifocal lung tumours in KP GEM mice. (B) KP cells in unifocal lung tumours. (C) CMT cells unifocal lung tumours and (D) LLC cells unifocal lung tumours.


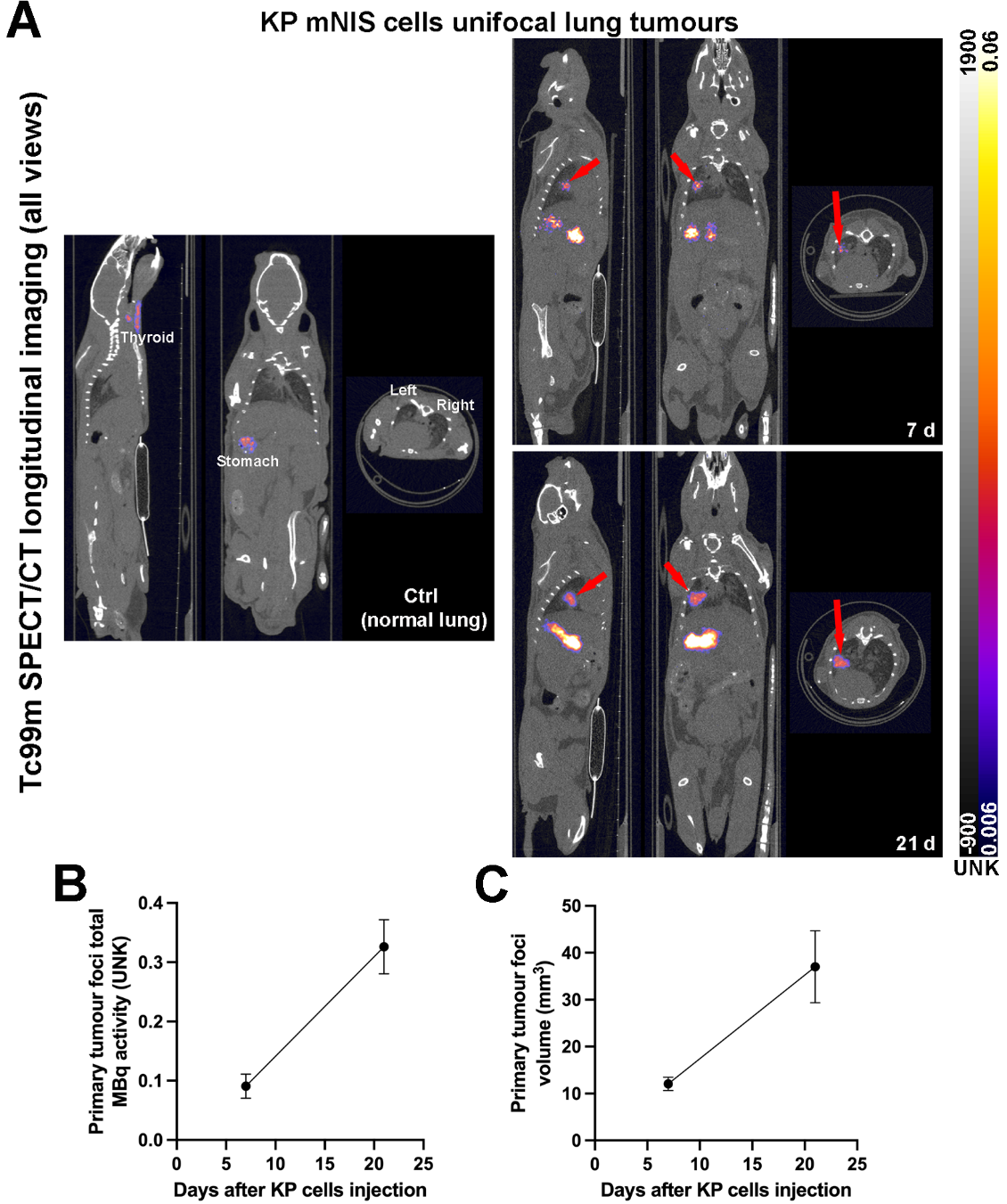


**Figure S4. *In vivo* SPECT/CT longitudinal imaging to track growth of left lung lobe primary KP cell tumour (extension to main Figure 2).** (A) Representative SPECT/CT overlaid images (all views) of time points after KP mNIS cell injection into left lung lobe. Left panel shows a control mouse (not injected with cells; Ctrl) showing no Tc99m uptake in the lungs (signal from stomach, thyroid, and bladder constitutes normal uptake of the radionuclide in these organs). *Red arrows*: primary tumour foci in the left lung lobe with high Tc99m uptake signal that increases over time. Scale bars for both CT and non-calibrated SPECT signals. (B) Primary tumour total MBq activity line graph represents total amount of Tc99m activity (UNK) in the total volume of primary tumour, over time. (C) Primary tumour volume line graph represents total volume (mm^3^) increase over time*. n =*4 mice/time point.

**
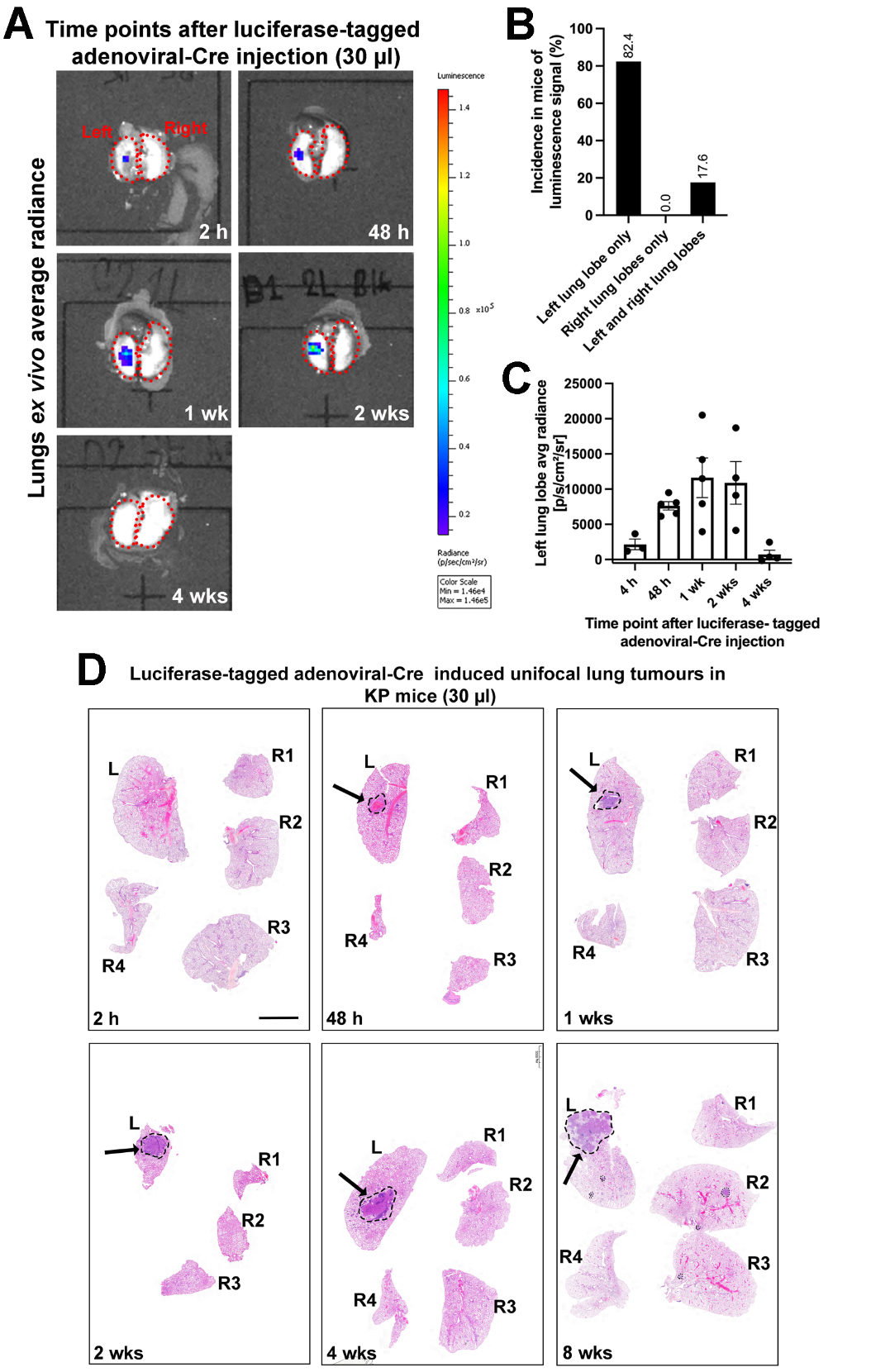
**

**Figure S5. Evidence of unifocal primary tumour in adenoviral-Cre luciferase (30****μl in standard concentration Matrigel) injected into KP mice.** (A) Luciferase-tagged adenoviral-Cre (1.25×10^7^ PFUs in standard concentration Matrigel in 30 μl injection volumes) was injected into the left lung lobe of KP GEM mice to generate a single primary tumour focus. Mice were culled and lungs imaged *ex vivo* using IVIS at 2–4 h, 48 h, 1, 2, and 4 weeks after injection to assess leakage from primary tumour injection site. Representative images of lungs dorsal view average radiance at different time points after injection. *Dotted red lines* represent the left and right lung sides, and intensity of radiance is seen in the left lung lobe according to scale on the right. (B) Bar graph denoting incidence in mice of luminescence signal detected in left lung lobe alone, right lung lobe alone, or both (considering both dorsal and ventral lung views), expressed as a percentage of total mice, combining all time points up to 2 weeks. *n =*17 mice. (C) Bar graph denotes left lung lobe average radiance (p/s/cm^2^/sr) calculated by summing the dorsal view radiance and ventral view radiance in the left lung lobe. Each dot represents a mouse. *n =*3 mice for 2–4 h; *n =*5 mice for 48 h; *n =*5 mice for 1 week; *n =*4 mice for 2 weeks; *n =*4 mice for 4 weeks. (D) Representative H&E-stained sections of lungs at progressive time points after adenoviral-Cre intralobular injection. *Dashed black lines and black arrows* highlight primary tumour in left lung lobe. Scale bars, 4 mm.

**
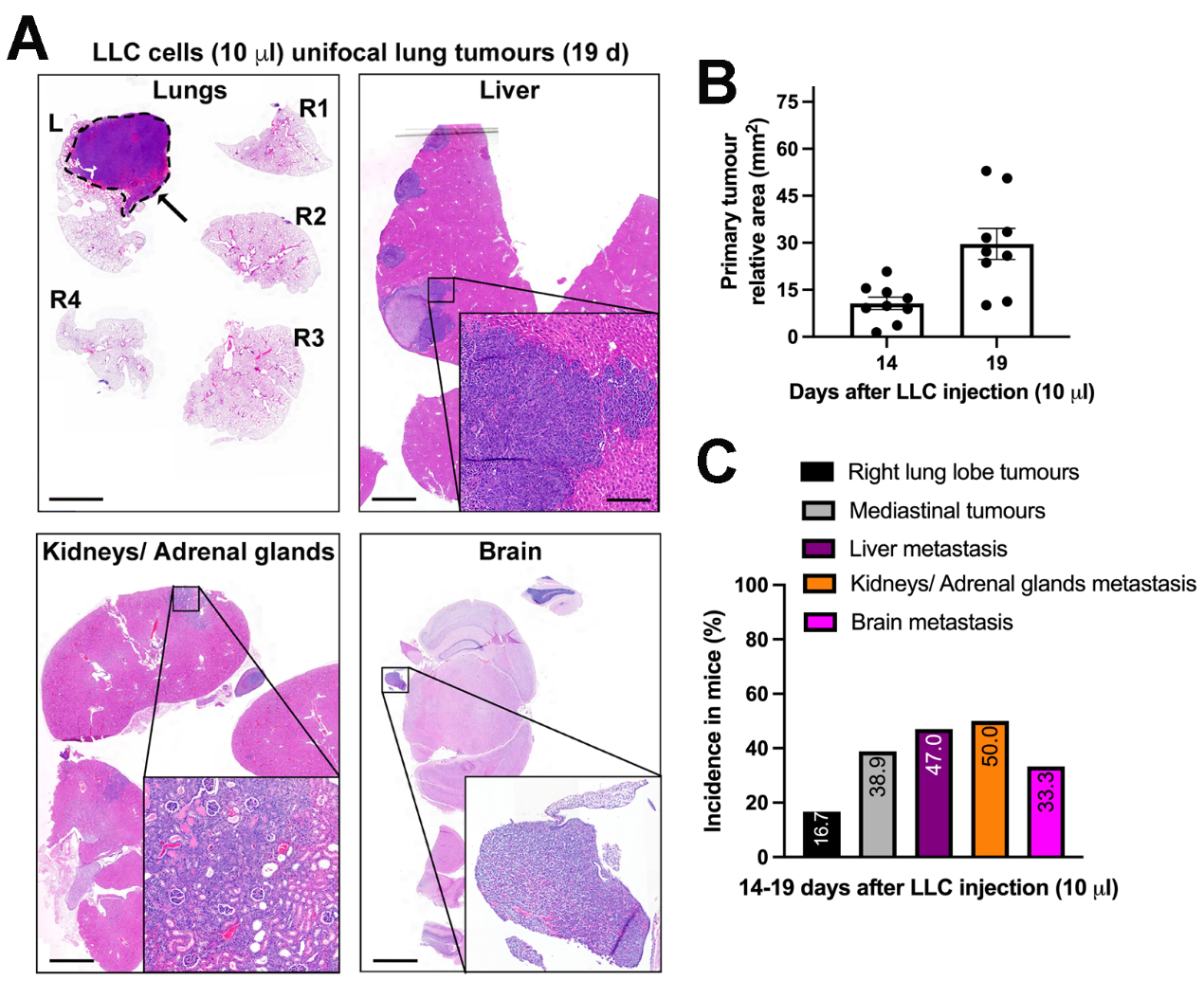
****Figure S6. Unifocal lung tumours induced by LLC cells injected (3,300 cells in 10 μl with standard concentration Matrigel) into C57Bl/6 mice.** (A) Representative lung, liver, kidneys, adrenal glands, and brain H&Es at 19 days after LLC cells injections (3,300 cells in 10 μl standard-concentration Matrigel). *Dashed black lines* and *arrows* highlight primary tumour foci in left lung lobe. *Black squares* denote metastasis that are shown in high power in the inserted panel. (B) Bar graph denoting primary tumour relative area in time courses after injection. *n =*9 mice for each time point. (C) Bar graph denoting incidence in mice of right lung lobe tumours (black bars), mediastinal tumours (grey bars), liver metastasis (purple bars), kidney and adrenal gland metastasis (orange bars), and brain metastasis (pink) expressed as a percentage of total mice 14–19 days after injection; *n =*18 mice, right lung lobe; *n =*17 mice, mediastinal; *n =*17 mice, liver; *n =*16 mice, kidney and adrenal glands; *n =*6 mice, brain. Scale bars, lungs at 4 mm; organs at 2 mm for low magnification and 0.2 mm for high magnification.

**
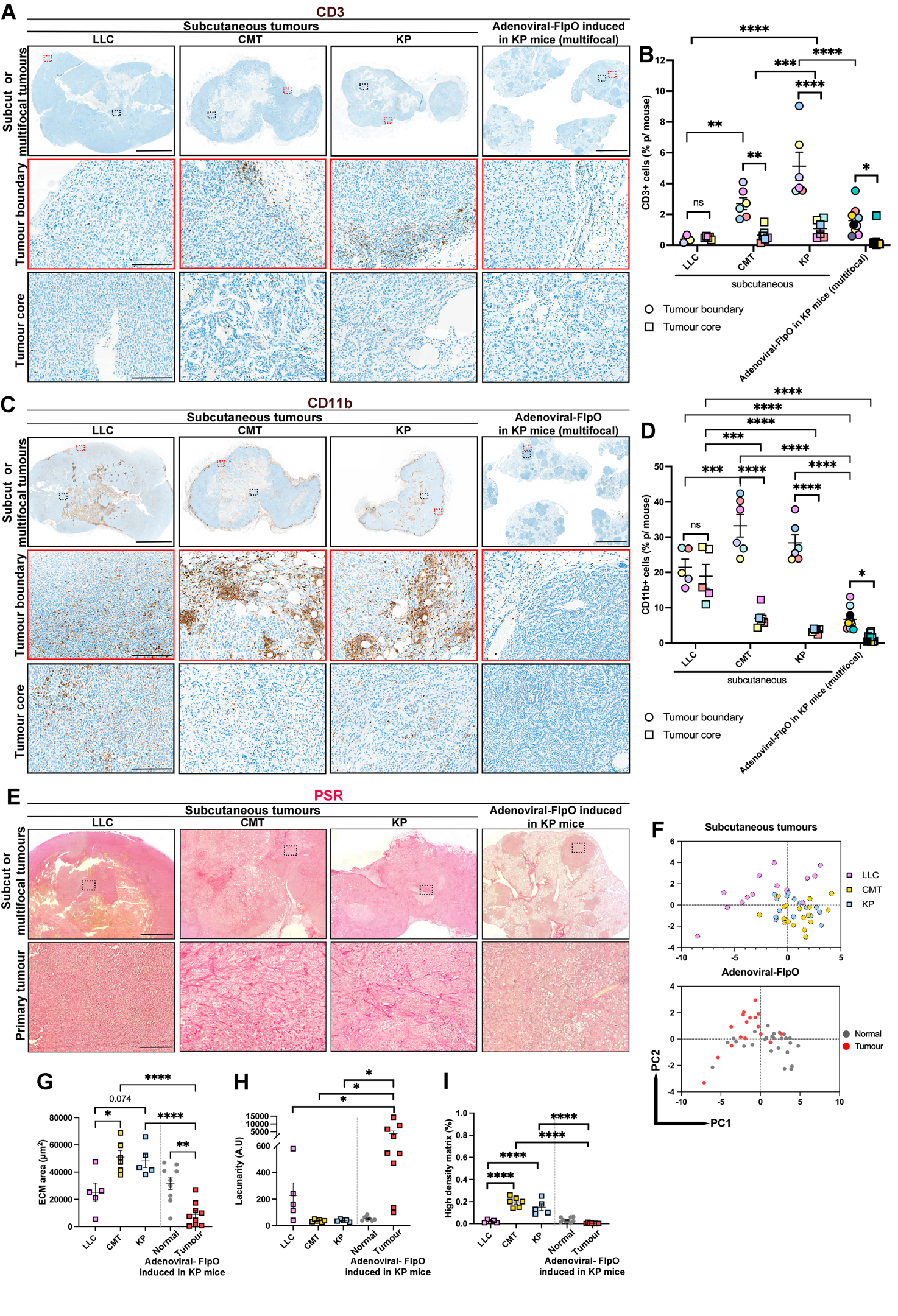
**

**Figure S7. Characterisation of lymphocytic and myeloid immune cell infiltration and extracellular matrix pattern of normal and tumour tissue in subcutaneous models for cell lines and multifocal adenoviral-induced in KP mice.** (A and C) Representative immunohistochemistry images of CD3 and CD11b (DAB with haematoxylin blue counterstain) respectively of subcutaneous tumours (in LLCs, CMTs, KP cells) and adenoviral-FlpO-induced multifocal tumours in KP GEM mice. Upper row: low-magnification images. *Black dashed boxes*: tumour core; *red dashed boxes*: tumour boundary and tumour core presented at higher magnification in lower rows. (B and D) Scatter dot plots showing percentage of CD3- and CD11b-positive cells, respectively. Each dot represents a subcutaneous tumour or an average of data from multiple foci of the multifocal model. Data given as mean ± SEM. LLC, *n =*5 mice; CMT, *n =*6 mice; KP, *n =*6 mice; multifocal adenoviral-FlpO in KP mice, *n =*9 mice. (E) Representative immunohistochemistry images of Picrosirius Red (PSR in red and Weigert’s haematoxylin in brown/orange), of subcutaneous tumours (for cell lines), and adenoviral-FlpO,induced multifocal tumours in KP GEM mice, denoting higher-magnification images (*dashed rectangles* show location of higher-magnification images in left lung lobe lower-magnification images). (F) PCA plots show overall ECM architecture in subcutaneous tumours (in LLC, CMT, KP) and between multifocal tumours (red dots) and their corresponding normal regions (grey dots) in adenoviral-FlpO in KP mice model. (G–I) Dot plots showing measurements: (G) ECM area (in μm^2^), (H) lacunarity (measure of how the ECM fills the space in arbitrary units), and (I) high-density matrix (as percentage) in the tumour compared with normal tissue. Each dot represents one mouse. LLC, *n =*5 mice; CMT, *n =*6 mice; KP, *n =*5 mice; multifocal adenoviral-FlpO in KP mice, *n =*9 mice. A two-way ANOVA with Tukey’s multiple comparisons test was used. ns, not significant; **p* ≤ 0.05; ***p* ≤ 0.01; ****p* ≤ 0.001; *****p* ≤ 0.001). *P* values are also given as numerical values for some comparisons. Scale bars, 2 mm at lower magnification (upper panels), 0.2 mm at higher magnification (middle and bottom panels).

**
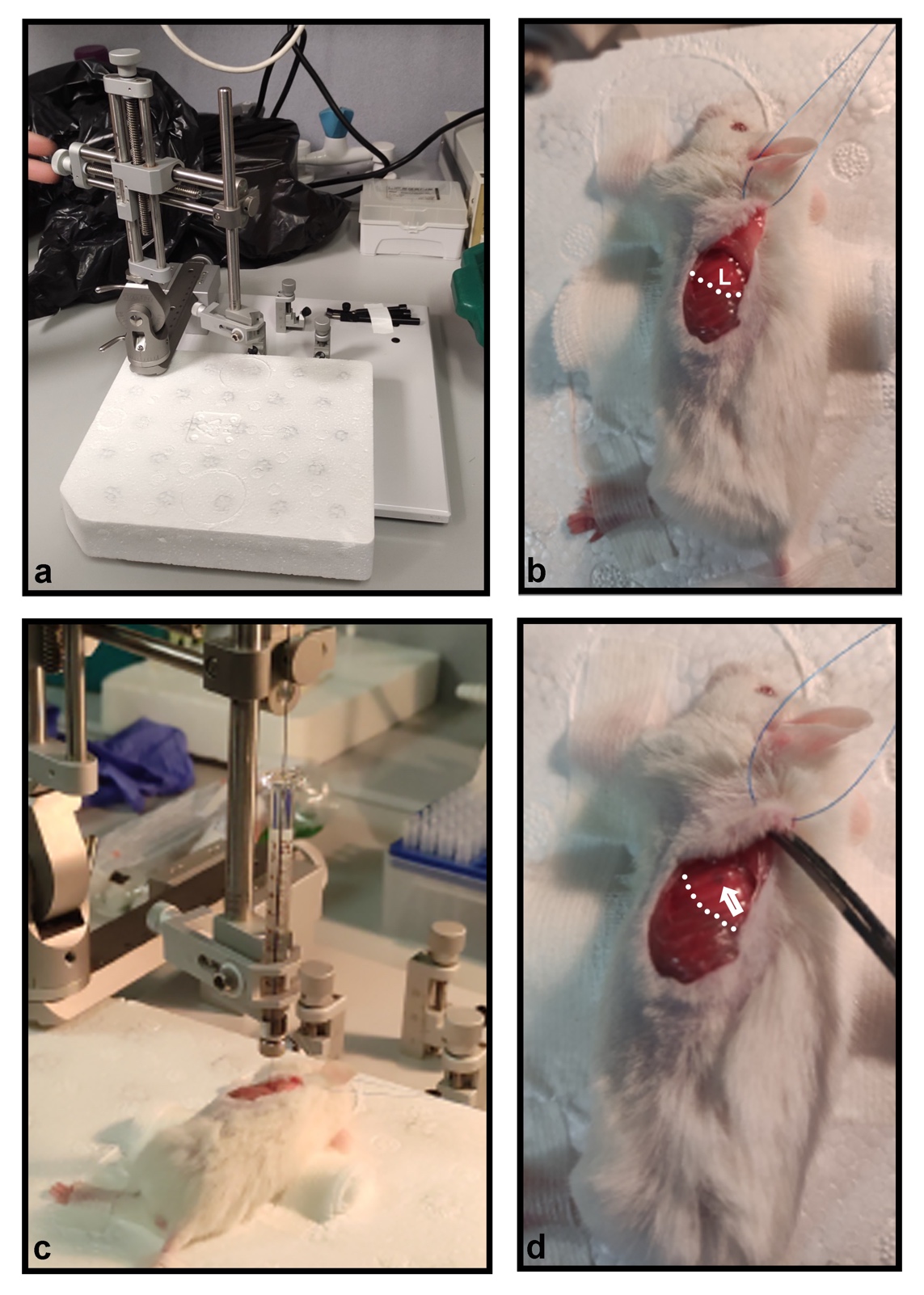
**

**Figure S8. Left lung lobe intralobular stereotactic injection procedure.** (A) Micro manipulator used for stereotactic injections. (B) Mouse is laid on its right side, with left side up and a rolled-up piece of gauze placed underneath to raise the thorax. A skin incision is made in left thorax to expose pleura with ribs and lung underneath (dotted white line denotes limit of lung). (C) The needle is placed above the injection site and inserted 3.5 mm into lung. (D) The 2 µl injected Matrigel/cell suspension stays localised in lung. The white arrow indicates injection area with Matrigel seen as darker spot in lung.

**Supplementary movies**

**Movies S1 and S2. Representative SPECT/CT maximum-intensity projection movie at 21 days (movie 1) and 35 days (movie 2) after LLC mNIS cell injection (10-μl injection volumes in high concentration Matrigel) into left lung lobe (extension to Figure 4).** Signal from stomach, thyroid, and bladder constitutes normal physiological uptake of radionuclide in these organs. Left lung lobe primary tumour foci in left lung lobe with high Tc99m uptake signal and small hot spots at level of abdomen highlight extra-thoracic metastasis. Scale, NIH fire min 0.006 and max 0.045 (SPECT signal) and min −900 and max 9,000 (CT signal).

**Table S3.** Summary of mutations identified in cell lines used and their relevance in NSCLC.

| **Mutation** | **Prevalence** | **Relevance** | **Targeted therapy** |
| --- | --- | --- | --- |
| ***KRAS*** | 21.2% | KRAS is a GTPase that acts as an upstream regulator of the MAPK pathway. The most common are codon 12 substitution mutations G12C (39%), G12V (18–21%), G12D (14–18%), and G12A (10–11%).  G12C is most common mutation (45%) in ex/current smokers, and G12D is most common mutation (46%) in never smokers. | G12C-targeted inhibitors sotorasib and adagrasib: FDA approved for treatment of adult patients with *KRAS* G12C-mutant NSCLC. |
| ***NRAS*** | 1.3% | NRAS is a GTPase | *NRAS* mutations and upregulation can confer resistance to cancer therapies, including EGFR and BRAF inhibitors |
| ***RET*** | 2.1% | RET is a receptor tyrosine kinase. Common mutation in RET are fusions which result in constitutive activation of RET kinase activity through dimerisation mediated by the RET fusion partner.  RET fusions have been shown to transform cell lines *in vitro* and promote lung tumourigenesis in mouse models | RET-targeted inhibitors selpercatinib and pralsetinib are FDA approved for the treatment of patients with metastatic RET fusion-positive NSCLC.  RET fusions have been shown to be sensitive to small-molecule inhibitors such as vandetanib and cabozantinib and are recognised by the FDA as predictive of response to inhibitors selpercatinib and pralsetinib. |
| ***PIK3CA*** | 6.1% | PIK3CA is the catalytic subunit of PI3 kinase | *PIK3CA* mutations after treatment with EGFR TKIs can lead to resistance to EGFR TKIs in NSCLC. |
| ***ALK*** | 3.4% | ALK, a receptor tyrosine kinase, is recurrently altered by chromosomal rearrangements | ALK-targeted inhibitors crizotinib, ceritinib, alectinib, brigatinib, lorlatinib, and ensartinib are FDA approved for treatment of ALK-positive lung cancer |
| ***BRAF*** | 4.1% | BRAF is an intracellular kinase. The V600E mutation deregulates kinase activity of protein and leads to constitutive BRAF activation | RAF-targeted inhibitors dabrafenib and encorafenib, in combination with MEK1/2-targeting inhibitors trametinib and binimetinib, respectively, are FDA approved for treatment of BRAF V600E mutant NSCLC. |
| ***MET*** | 4.4% | MET is a receptor tyrosine kinase. MET D1010 mutation is known to be oncogenic | MET-targeted inhibitors capmatinib and tepotinib FDA-approved for the treatment of patients with metastatic NSCLC harbouring *MET* exon 14 skipping mutations |
| ***STK11*** | 10% | STK11 is a tumour suppressor and intracellular kinase | STK11-altered NSCLC may be sensitive to AXL inhibitor bemcentinib in combination with anti-PD-1 antibody pembrolizumab. |
| ***DDR2*** | 2.9% | DDR2 is a receptor tyrosine kinase. Somatic gain-of-function mutations in DDR2 have been identified in squamous cell lung cancers | Clinical responses to targeted therapy with dasatinib have been reported in patients with squamous cell lung cancer with *DDR2* mutations |
| ***CTNNB1*** | 3.5% | CTNNB1 is a transcriptional activator | Cancers with *CTNNB1* mutations are thought to be resistant to pharmacological inhibition of upstream components of WNT pathway and instead require direct inhibition of β-catenin function |
| ***ATM*** | 7.1% | ATM is a kinase involved in DNA damage response. ATM mutations are positively associated with female gender, smoking history and non-squamous histology, TMB, and PD-L1 positivity. | *ATM*-mutant cancers are increasingly sensitive to DNA-damaging agents due to deficiencies in DNA repair pathways.  Loss of ATM may result in a better response to checkpoint inhibition. |
| ***RB1*** | 5.3% | RB1 is a regulator of the cell cycle.  Mutations include loss of function and deletions of RB1, and concomitant loss of RB1 and p53 is thought to be a tumour-initiating event. *RB1* mutation status has been found to be strongly associated with poorer outcomes in NSCLC | Not FDA-approved therapy |
| ***TP53*** | 52.7% | TP53 is a tumour suppressor in DNA damage pathway. TP53 alterations in NSCLC have a worse prognosis and may be more resistant to chemotherapy and radiation | There are promising clinical data in patients with *TP53* Y220C mutant solid tumours treated with TP53 structural corrector rezatapopt |
| ***CDKN2A*** | 5.6% | The *CDKN2A* gene encodes two proteins, p16INK4A and p14ARF, which regulate cell growth and survival. The role of *CDKN2A* loss of function in ICB resistance in NSCLC is controversial | Cancer cells with loss of function of *CDKN2A* may be sensitive to CDK4/6 inhibitors such as palbociclib, ribociclib and abemaciclib |

Data extracted from cBioPortal for Cancer Genomics (<http://cbioportal.org>) and OncoKB (<https://www.oncokb.org/>). Date last accessed 3 February 2025.

**Table S4.** Summary of features of lung cancer unifocal models presented in this study.

| **Parameters/ Model** | **LLC**  **cells** | **CMT**  **cells** | **KP**  **cells** | **Adenoviral- induced in KP mice** |
| --- | --- | --- | --- | --- |
| **Mutations** | *Kras* G12C  *Nras* Q61H  *Pik3ca* LOF  *Alk* Q1068K  *Braf* L262Q  *Met* I851M  *Stk11* S13T  *Ddr2* S673G  *Ctnnb1* G235S  *Cdkn2a* LOF | *Kras* G12V  *Ret* R818H  *Rb1* | *Kras* G12D  *Trp53* LOF  *Ret* A877T  *Pik3ca* I273M  *Alk*  *Atm* | *Kras* G12D  *Trp53* LOF |
| **Survival** | 30 μl, up to 21–26 days  10 μl, up to 35 days | 30 μl, up to 35-45 days  10 μl, not tested | 30 μl, up to 35-45 days  10 μl, not tested | 30 μl, up to 16 weeks  10 μl, up to 12 weeks |
| **Lung secondary tumours** | Yes | Yes | Yes | Yes |
| **Mediastinal tumours** | Yes | Yes | Yes | No |
| **Long distance metastasis**  **(% of mice)** | Liver, 75%;  kidneys/adrenal glands, 70%; brain, 20% (only observed in 2/6 with 10 μl normal conc. Matrigel) | Not tested | Not tested | No  Up to 12–16 weeks |
| **Lymphocytic infiltration** | Very low/absent | Present (boundary) | Present  (boundary) | Present  (boundary and core) |
| **Myeloid infiltration** | Present (highest in boundary) | Present (boundary and core) | Present (boundary)  Low (core) | Low  (boundary and core) |
| **Matrix remodelling (tumour versus normal surrounding tissue)** | No  (no difference) | No  (increased HDM) | Yes  (increased HDM) | Yes  (increased ECM area and HDM, decreased lacunarity) |
| **Histopathological features** | Necrosis  STAS  Vascular invasion  PT | Necrosis | Necrosis  STAS  Vascular invasion | Necrosis  STAS  Vascular invasion  GTC |
