# Supplementary figures and images for: Development and characterisation of improved unifocal primary mouse lung cancer models with metastatic potential
Source: J Pathol. 2025 Jun 18;266(4-5):405–20. doi: 10.1002/path.6435 (PMC12256383; doi:10.1002/path.6435)

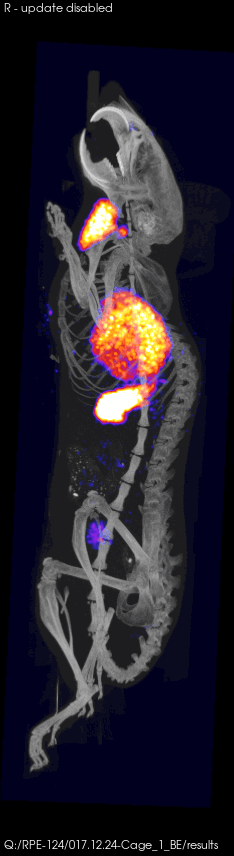

Supplement: Supplementary file 2 — Movie S1. Representative SPECT/CT maximum‐intensity projection movie at 21 days after LLC mNIS cell injection (10‐μl injection volumes in high‐concentration Matrigel) into left lung lobe (extension to Figure 4) [file PATH-266-405-s002.gif]

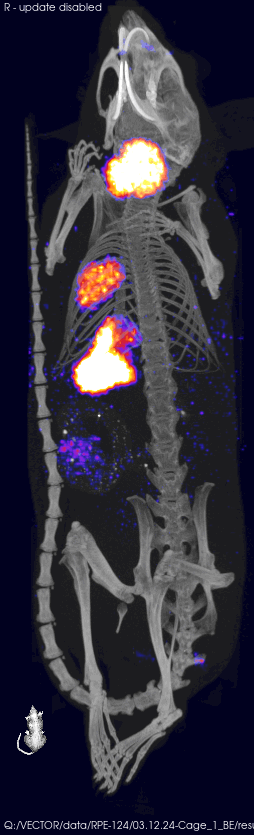

Supplement: Supplementary file 3 — Movie S2. Representative SPECT/CT maximum‐intensity projection movie at 35 days after LLC mNIS cell injection (10‐μl injection volumes in high‐concentration Matrigel) into left lung lobe (extension to Figure 4) [file PATH-266-405-s003.gif]
